# Purified fibers in chemically defined synthetic diets destabilize the gut microbiome of an omnivorous insect model

**DOI:** 10.3389/frmbi.2024.1477521

**Published:** 2024-12-12

**Authors:** Rachel Louise Dockman, Elizabeth A. Ottesen

**Affiliations:** Department of Microbiology, University of Georgia, Athens, GA, United States

**Keywords:** gut microbiome, cockroach (*Periplaneta americana*), fiber, diet, xylan, whole food, processed food

## Abstract

The macronutrient composition of a host’s diet shapes its gut microbial community, with dietary fiber in particular escaping host digestion to serve as a potent carbon source for gut microbiota. Despite widespread recognition of fiber’s importance to microbiome health, nutritional research often fails to differentiate hyper-processed fibers from cell-matrix-derived intrinsic fibers, limiting our understanding of how individual polysaccharides influence the gut community. We use the American cockroach (*Periplaneta americana*) as a model system to dissect the response of complex gut microbial communities to dietary modifications that are difficult to test in traditional host models. Here, we designed synthetic diets from lab-grade, purified ingredients to identify how the cockroach microbiome responds to six different carbohydrates (chitin, methylcellulose, microcrystalline cellulose, pectin, starch, and xylan) in otherwise balanced diets. We show via 16S rRNA gene profiling that these synthetic diets reduce bacterial diversity and alter the phylogenetic composition of cockroach gut microbiota in a fiber-dependent manner, regardless of the vitamin and protein content of the diet. Comparisons with cockroaches fed whole-food diets reveal that synthetic diets induce blooms in common cockroach-associated taxa and subsequently fragment previously stable microbial correlation networks. Our research leverages an unconventional microbiome model system and customizable lab-grade artificial diets to shed light on how purified polysaccharides, as opposed to nutritionally complex intrinsic fibers, exert substantial influence over a normally stable gut community.

## 1 Introduction

The gut microbiome is a key player in host metabolism and homeostasis; it extracts energy from recalcitrant dietary components, provisions essential nutrients, and stimulates the host’s immune system to protect against pathogens and toxins (Bäckhed et al., 2005; Huttenhower et al., 2012; Thaiss et al., 2016; Belkaid and Harrison, 2017). These benefits to the host are contingent upon the microbiota present, which themselves are selected through external pressure such as host genetics environment, and diet (Goodrich et al., 2014; Sonnenburg et al., 2016; Kurilshikov et al., 2017; Greene et al., 2020; Kurilshikov et al., 2021). Diet has gained particular attention as the most easily manipulated of these factors, and a clear relationship exists between microbially derived metabolic products from the gut microbiome and overall host health (Tanes et al., 2021; Wolter et al., 2021).

Shifts in the ratios and sources of metabolizable macronutrients (fats, carbohydrates, and protein) are frequently identified as drivers of diet-associated microbiota alterations, but the most important component to resident gut bacteria is what bypasses host digestion relatively untouched: fiber (Walker et al., 2011; Rastall et al., 2022). Dietary fiber consists of plant-derived structural carbohydrates that most animals are unable to process and are thus key to maintaining a diverse, beneficial gut microbial community. However, performing research relating dietary fiber consumption to gut microbiota within a host organism presents several challenges. Whole foods contain “intrinsic fibers”, an assortment of carbohydrates characterized by source-specific molecular structures that form close associations with plant proteins and cell-matrix components (Augustin et al., 2020; Tuncil et al., 2020; Puhlmann and de Vos, 2022). These heterogeneous structures can obscure the influence of individual polysaccharides on the gut community, especially considering the high diversity of carbohydrate-degrading machinery found across individual lineages of gut microbiota (Kaur et al., 2019; Liu et al., 2020b; Villa et al., 2020). Purified fibers present an alternative that controls for these variable compounds, but mammalian models have complex nutritional needs that limit the extent of dietary manipulation possible before introducing host stresses. As a result, *in vivo* dietary research on fiber-microbiome dynamics frequently uses balanced diets containing host-metabolizable carbohydrates that are supplemented with purified fibers, therefore exposing gut bacteria to a mix of carbon sources. This restricts the conclusions that can be drawn from the microbial response to the fiber itself, since there is no way to prevent gut microbiota from prioritizing an alternative energy source instead of the fiber of interest. Invertebrate models offer more flexibility, but well-known insect models such as *Drosophila* have a limited dietary range that poorly reflect the community dynamics found in mammalian host species (Lee and Brey, 2013; Lesperance and Broderick, 2020). To address this challenge, we are developing the omnivorous American cockroach (*Periplaneta americana*) as a model of microbiome dynamics that extends our understanding of human-relevant bacteria while leveraging the benefits of invertebrate research.

The cockroach digestive system is divided into three major regions: the foregut, midgut, and hindgut. The foregut, analogous to the mammalian stomach, consists of a large crop where salivary amylase and midgut-derived trypsin initiate the digestion of carbohydrates and proteins prior to mechanical breakdown via the proventriculus or gizzard (Tamaki et al., 2014). The midgut, analogous to the mammalian small intestine, is lined with a continuously secreted chitinous peritrophic matrix (rather than a mucus layer) and serves as the primary source of host-derived aminopeptidases, cellobiase, and lipase, as well as the primary site of nutrient absorption (Eisner, 1955; Tamaki et al., 2014). The hindgut, analogous to the mammalian large intestine, facilitates microbial fermentation of undigested and/or unabsorbed dietary substrates (Engel and Moran, 2013). Fiber comprises most of this undigested material, but as in mammals, other macronutrients may escape host digestion due to factors such as plant-derived protease inhibitors or the diet’s structural complexity (Ryan, 1990; Engel and Moran, 2013).

Despite the obvious differences between cockroaches and humans, in the context of host-microbe symbioses, there are both similarities and unique benefits that support the use of omnivorous cockroaches as a promising model for studying diet-gut microbiome dynamics. American cockroaches are colonized by a complex hindgut microbiome that is taxonomically similar to the human colonic flora, consisting of many shared family and genus-level microbial lineages within the Bacteroidota, Firmicutes (now Bacillota), and Proteobacteria (now Pseudomonadota) (Cruden and Markovetz, 1987; Schauer et al., 2012; Tinker and Ottesen, 2016). These microbiota also play functionally analogous roles to their mammalian counterparts in host nutrition, with hindgut bacteria scavenging escaped nutrients and fermenting otherwise indigestible dietary components into volatile fatty acids that are absorbed by the host for energy (Zurek and Keddie, 1996; Ayayee et al., 2018; Jahnes and Sabree, 2020; Vera-Ponce de León et al., 2021). Further, cockroaches host in their fat body an endosymbiotic bacterium, *Blattabacterium*, that protects the host from short-term starvation through the conversion of stored uric acid into essential amino acids (Cochran et al., 1979; Sabree et al., 2009; Ayayee et al., 2016). This unique trait enables the cockroach to survive extreme dietary manipulation for extended periods of time.

Studies of cockroach gut microbiome responses to diet have generated contrasting responses, with multiple large-scale studies finding that the cockroach gut microbiome is highly stable between groups given differing diets (Schauer et al., 2014; Tinker and Ottesen, 2016; Lampert et al., 2019), while others have demonstrated that diet alterations result in different gut microbiome configurations (Bertino-Grimaldi et al., 2013; Pérez-Cobas et al., 2015; Zhu et al., 2022). Currently, there is no consensus on why these studies produced differing results and comparison is difficult due to the inconsistent use of synthetic or whole-food diets across studies. Structurally complex whole foods may obscure bacterial responses to specific nutritional alterations, but synthetic diets are amenable to precise dietary changes, thus allowing stricter variable control (Okarter and Liu, 2010; Koropatkin et al., 2012; Tuncil et al., 2017). Artificial diets have been successfully developed for insects with far more specialized dietary needs than cockroaches, suggesting that cockroaches are ideal candidates for dietary experimentation with lab-synthesized diets (Piper et al., 2014; Talyuli et al., 2015; Gonzales et al., 2018; Majumder et al., 2020).

To facilitate precise manipulation of dietary composition in cockroaches, we have developed a series of synthetic cockroach diets based on the work of early entomologists (Noland et al., 1949; Haydak, 1953; Hamilton and Schal, 1988). These artificial diets serve as a nutritionally complete base to isolate the influence of specific dietary components on the *P. americana* hindgut microbiome, a community known to be resistant to dietary manipulation when fed macronutrient-biased whole-food diets (Tinker and Ottesen, 2016). Using these synthetic diets as a base, we tested a spectrum of purified polysaccharides as the primary carbon and energy source to identify whether the hindgut microbiome responds to specific fibers without obfuscation by intrinsic fiber components. We found that these diets resulted in much stronger impacts on gut microbiome composition than highly divergent whole-food diets, with long-chain polysaccharide sources exerting the largest effect despite alterations in their protein and micronutrient composition. Our work will facilitate future studies of gut microbiome responses to fine-scale dietary composition in cockroaches and shed light on how hyper-processed synthetic diets, which superficially appear to be nutritionally complete, destabilize a complex gut microbiome.

## 2 Materials and methods

### 2.1 Insects and experimental conditions

Our *P. americana* colony has been maintained in captivity at the University of Georgia for over a decade. Mixed age and sex stock insects are maintained at room temperature in glass aquarium tanks with wood chip bedding and cardboard tubes for shelter in a 12:12 light:dark cycle. Water via a cellulose sponge fit to a Tupperware reservoir and dog food [Purina ONE chicken & rice formula, guaranteed analysis: 26% crude protein (min), 16% crude fat (min), 3% crude fiber (max)] are provided to the stock colonies *ad libitum*.

### 2.2 Synthetic diets

The synthetic diets created for dietary testing were designed to provide balanced nutrition while remaining malleable to component manipulation. The diets contained Vanderzant vitamin mix (catalog #: 903244, MP Biomedicals, Irvine, CA, USA), Wesson salt mix (catalog #: 902851, MP Biomedicals), peptone (catalog #: J636, Amresco, VWR International, Radnor, PA, USA), casein (catalog #: C3400, Sigma-Aldrich, St. Louis, MO, USA), and cholesterol (catalog #: 0433, VWR); the amounts are listed in **Table 1**. The dry ingredients were suspended in sufficient volumes of diH_2_O to create a batter or dough, formed into pellets, and then dehydrated at 65°C until they were sufficiently dry to maintain shape. Food pellets were stored at -20°C until use.

**Table 1:** Synthetic diet compositions.

Diet Type	CHO %	Casein %	Peptone %	Mineral Mix %	Vitamin Mix %	Cholesterol %	Diet/CHO
Standard: Polysaccharide	70.5	17	8	3	0.5	1	Chitin, MeC, MCC, Pectin, Starch, Xylan
Standard: Simple Sugar	70.5	17	8	3	0.5	1	Cellobiose, Glucose, Xylose
Protein Deficient	95.5	0	0	3	0.5	1	MCC P-, Xylan P-
Vitamin Deficient	72	17	8	3	0	0	MCC V-, Xylan V-
Tuna-Amended	70.5	25% tuna		3	0.5	1	MCC, Xylan

CHO, carbohydrate; MeC, methylcellulose; MCC, microcrystalline cellulose; P-, protein deficient; V-, vitamin deficient; *: canned tuna was dried prior to weighing.

In most experiments, the only component changed was the carbohydrate source. The polysaccharides used include microcrystalline cellulose (MCC with 51um particle size; catalog #: 435236, Sigma-Aldrich), methylcellulose (catalog #: M0512, Sigma-Aldrich), xylan from corn core (catalog # TCX0078, TCI Chemicals, Portland, OR, USA), pectin from apple (catalog # 93854, Sigma-Aldrich), starch from potato (catalog #: S516, Fisher Chemical), and chitin (catalog #: J61206, Alfa Aesar, Ward Hill, MA, USA). For simple sugar diet variations, cellobiose (catalog #: 22150, Sigma-Aldrich), glucose (catalog #: G7021, Sigma-Aldrich), and xylose (catalog #: 200001-008, Acros Organics, VWR International) were used as the carbohydrate component.

### 2.3 Experimental design

Experimental conditions were prepared as described in Tinker and Ottesen (2016). Briefly, mixed-sex healthy adult insects (n=12/diet) were transferred from the stock colony to plastic tanks containing pebbles and bleached polyvinyl chloride tubes for footing and shelter, respectively. Food and water were provided *ad libitum* in rigid plastic or glass dishes following two days of food restriction and habituation. Dietary treatments for the four cohorts (**Supplementary Table 1**) lasted 2 weeks, during which debris, oothecae, and lethargic insects were removed daily.

Upon completion of dietary treatments, all insects were sacrificed for sample collection. Insects were isolated in a sterile culture plate and placed on ice until torpid, upon which they were decapitated and dissected. Sternites were removed with sterile forceps to expose the intact gut and fat body tissue was cleaned away. The cleaned gut was frozen on a sterile aluminum dish on dry ice and divided into foregut, midgut, and hindgut sections for collection in 500-800µL phosphate-buffered saline (1X PBS). Gut contents and tissue-attached bacteria were disrupted with a sterile pestle, and the samples stored at –20°C until DNA extraction. For this study, only the hindgut community was analyzed due to its higher microbial density and activity than in other gut regions.

### 2.4 DNA extraction

DNA was extracted from 200µL aliquots of all individual samples using the EZNA Bacterial DNA Kit (Omega Biotek, Norcross, GA, USA) with some modifications. Sample aliquots were centrifuged at 5000g for 10min, with the resulting pellet resuspended in 100µL TE buffer plus 10µL lysozyme (50mg/mL) and incubated for 30 min at 37°C. Following incubation, samples were vortexed with glass beads (25mg, Omega Biotek) for 5 min at 3000rpm, then incubated at 55°C for 1 hour with 100µL TL buffer, 20µL proteinase K, and continuous 600rpm shaking. The kit protocol was followed for additional incubations with BL buffer and DNA isolation using the provided column. DNA was eluted into 50µL of the provided Elution Buffer and quantified using either a Nanodrop Lite spectrophotometer (Thermo Scientific) or the Take3 plate for BioTek plate readers (Agilent).

### 2.5 16S rRNA gene library preparation and sequencing

The V4 region of the 16S rRNA gene was amplified via a 2-step polymerase chain reaction (PCR) from individual hindgut lumen samples (n=8-12/diet) as previously described in (Tinker and Ottesen, 2016; Tinker and Ottesen, 2020; Tinker and Ottesen, 2021). Both PCR reactions used 0.02U/L Q5 Hot Start high-fidelity DNA polymerase (New England BioLabs, Ipswich, MA, USA) with 200µM dNTPs and 0.5µM forward and reverse primers in 1M Q5 reaction buffer. The first 10µL reaction containing 3ng DNA and primers targeting the V4 region (515F: GTGCCAGCMGCCGCGGTAA; 806R: GGACTACHVGGGTWTCTAAT) was performed under the following conditions: activation at 98°C for 30s; 15 cycles of 98°C for 10s, 52°C for 30s, and 72°C for 30s; final extension at 72°C for 2 min. Immediately following amplification, 9µL of the first reaction was added to 21µL of Q5 reaction mix containing barcoded primers with adaptor sequences for Illumina sequencing (Caporaso et al., 2011). Cycling was performed as follows: activation at 98°C for 30s; 4 cycles of 98°C for 10s, 52°C for 10s, and 72°C for 30s; 6 cycles of 98°C for 10s and 72°C for 1 min; final extension at 72°C for 2 min.

After product size verification via gel electrophoresis, samples were cleaned as instructed in Omega Biotek’s Cycle Pure kit, quantified, and pooled for equimolar representation of each sample. Prepared libraries were sent to the Georgia Genomics and Bioinformatics Core at the University of Georgia for 250 base pair paired-end Illumina MiSeq sequencing.

### 2.6 Amplicon sequence variant generation

Each dataset collected in this study was processed separately in R (version 4.2.1) by sequencing run using R package DADA2 (version 1.24.0), with the cumulative Amplicon Sequence Variants (ASVs) generated input as a priors table for each successive run (Callahan et al., 2016; RStudioTeam, 2020). To allow for comparison with this dataset, raw data from previous research in the Ottesen lab were reprocessed to generate ASVs following the same procedures as in this current study (Tinker and Ottesen, 2016). All sequence tables produced by these datasets were combined by ASV sequence prior to taxonomy assignment to ensure continuity in naming. Taxonomy was assigned using DADA2 and the ARB Silva v138 classifier to the species level, uniquely numbered, and filtered to remove sequences matching eukaryotic (chloroplast, mitochondria) or endosymbiotic *Blattabacterium* DNA (Quast et al., 2013; Callahan et al., 2016).

### 2.7 Community analysis

Alpha and beta diversity analyses were performed via the R package vegan (version 2.6-4) (Oksanen et al., 2019). Samples were rarefied prior to diversity analysis to 7,924 reads for comparisons between synthetic and/or whole-food diets, 9,685 reads for analysis of repeat xylan and MCC diet experiments, and 12,274 reads for follow-up experiments exploring nutrient deficiencies and simple sugar carbohydrates. Alpha diversity was measured via the Shannon index, the count of ASVs observed in rarefied samples, and Pielou’s evenness (calculated as Shannon/log(Observed)). Weighted (binary = FALSE) and unweighted (binary = TRUE) Bray–Curtis dissimilarities were calculated, assessed for dispersion, and plotted using the vegan functions vegdist, betadisper, and metaMDS. Unweighted Bray–Curtis dissimilarity, or incidence-based Bray–Curtis dissimilarity, is equivalent to the Sørensen index in that it is based on the number of species shared or unique between groups without accounting for individual species abundance (Legendre and De Cáceres, 2013). Statistics for alpha diversity indices were calculated with the Wilcoxon rank sum test (pairwise comparisons) and the Kruskal–Wallis test (multi-group comparisons). The significance of community composition differences observed in beta diversity measures was assessed using PERMANOVA (vegan::adonis2()). Beta dispersion was further examined through Tukey’s HSD test for pairwise comparisons and ANOVA for multi-group comparisons.

Differential abundance analysis was conducted using DESeq2 (version 1.36) (Love et al., 2014). For identification of ‘diet-characteristic taxa”, raw count data for the synthetic diet set (n=66) were filtered to exclude ASVs present in fewer than five samples and run through the ‘DESeq’ command with parameters ‘fitType = “local”‘ and ‘design = ~ Diet’. Pairwise result tables were obtained for all diet comparisons and filtered for significant data, defined as having an adjusted p-value smaller than 0.05 and a baseMean larger than 10. ASVs significantly upregulated for one diet vs the other five diets were identified as diet-characteristic (n=76) and used to generate the heatmap in **Supplementary Figure S3**. For comparison of “synthetic vs whole”, raw count data for both diet sets (n=125) were combined and filtered to exclude ASVs that appeared in fewer than five samples. DESeq2 was run with parameters ‘fitType = “local”‘ and ‘design = ~ Diet_Type’ to identify differentially abundant ASVs between the diet types. The resulting baseMean and log2 fold change were used to generate the MA plots.

UpSetR (version 1.4) was employed to visualize intersecting sets of taxa, providing insights into the distribution of taxonomic features across samples (Conway et al., 2017). For UpSet analysis, samples were rarefied to 7,924 reads and then collapsed together to obtain total counts per diet. Both a presence/absence table and a proportion table were generated from these data, with the presence/absence table used for UpSet graph generation. The relative abundance of each set was calculated using the proportion table with ASVs collapsed per set and visualized as pie charts within the UpSet graph.

Co-correlation analysis was conducted to evaluate the impact of synthetic diets on microbial interaction networks using the SparCC procedure (Friedman and Alm, 2012). Networks were constructed separately for the synthetic diet group and the whole-food diet group using sequence count tables that were filtered to only include ASVs with at least five representatives present in 25% of samples (synthetic: 17 samples; whole food: 15 samples), preventing spurious correlations from rare taxa. SparCC was implemented in R with standard parameters, and the resultant networks were characterized and analyzed with the igraph R package (version 1.5.1) (Csardi and Nepusz, 2006; Csárdi et al., 2024). Networks were pruned to contain only edges with a correlation absolute value of at least 0.4 and exported into Cytoscape for visualization using the edge-weighted spring-embedded layout method (Shannon et al., 2003).

## 3 Results

### 3.1 Impacts of synthetic diets on gut microbiome diversity and community composition

We formulated a series of synthetic diets composed of a fixed base of 25% protein amended with dietary salts, vitamins, and cholesterol while differing only in complex carbohydrate type. Initial experiments utilized five alternative polysaccharide sources: chitin, methylcellulose, MCC, pectin, or xylan. Following the initial analysis of these results, we tested an additional starch-based diet. The prepared diets were readily consumed by the cockroaches in all cases.

**Figure 1:**
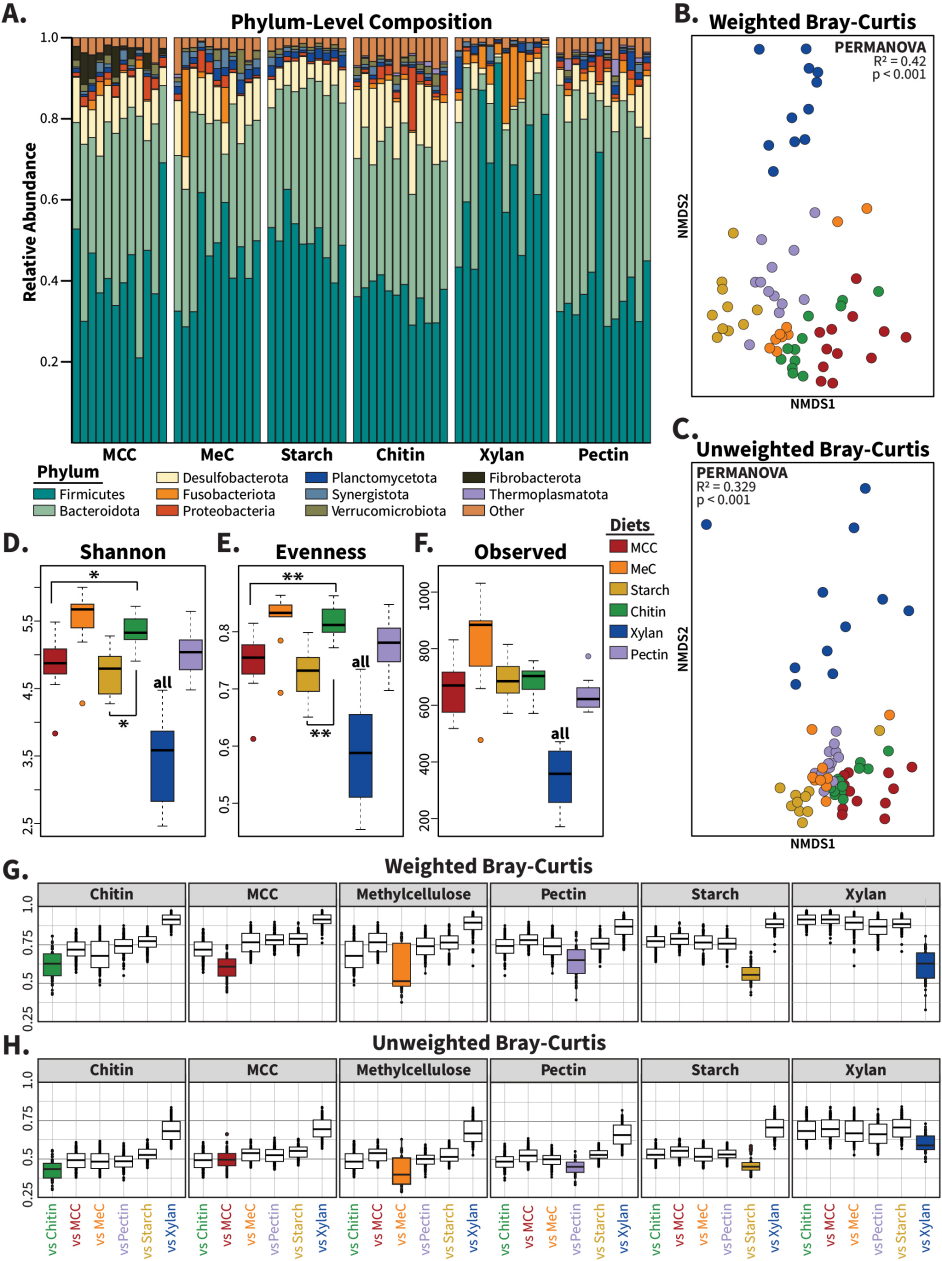
Composition of gut microbiomes from cockroaches fed synthetic diets. **(A)** Barplot showing the relative abundance of phyla across samples for each of the synthetic carbohydrate diets. Bars represent individual hindgut samples, clustered and labeled by diet polysaccharide source. Phyla present at an abundance greater than 1% in at least one sample are plotted. Non-metric multidimensional scaling (NMDS) was used to plot **(B)** weighted and **(C)** unweighted Bray–Curtis dissimilarity, with one point representing the community of one insect. The alpha diversity measures **(D)** the Shannon index, **(E)** Pielou’s evenness, and **(F)** the number of observed taxa were plotted. Boxplots of **(G)** weighted and **(H)** unweighted Bray–Curtis dissimilarity display each diet vs self (colored boxes) and the other five synthetic diets (white boxes). Samples were rarefied to a constant depth of 7,924 sequences for the alpha and beta diversity calculations. For alpha diversity measures, pairwise statistics were calculated with Wilcoxon rank-sum tests and multivariate analysis was performed using Kruskal–Wallis tests. PERMANOVA was used to generate statistics for ordination analyses. “all” indicates p<0.05 vs all other diets; * = p<0.05; ** = p<0.01.

To evaluate the impact of these diets on the gut community, each diet was fed to adult cockroaches (n=12/diet) for a period of 14 consecutive days, after which the insects were sacrificed, and their hindgut dissected out for 16S rRNA gene library sequencing. Following library preparation and sequencing, we used DADA2 to obtain 2,321,848 quality-controlled, assembled sequences assigned to 3,308 ASVs after the removal of endosymbiont (*Blattabacterium* sp.) and mitochondrial sequences (Callahan et al., 2016). At the phylum level, at least 80% of each sample was dominated by Bacteroidota, Firmicutes, and Desulfobacterota, in agreement with previous studies on the cockroach gut microbiome (**Figure 1A**) (Tinker and Ottesen, 2016; Tinker and Ottesen, 2020; Dukes et al., 2023). The relative abundances of these three phyla were similar across all samples excluding xylan-fed cockroaches; these insects hosted notably more Firmicutes and less Desulfobacterota than cockroaches fed other diets (**Figure 1A**).

Alpha diversity, as measured by the Shannon index, evenness, and community richness, significantly differed across diet treatments (**Figures 1D, F**; Kruskal Wallis p<0.001 for each). Pairwise analyses found that chitin-fed insects possessed higher Shannon index values (p<0.05) and community evenness (p<0.01) than those of MCC- and starch-fed insects, while the xylan diet resulted in lower alpha diversity measures than all other diets (p<0.05 for each).

Beta diversity analyses using weighted and unweighted Bray Curtis dissimilarity, which is also known as the Sørensen index, revealed significant impacts of our synthetic diets on gut microbiome composition. On average, between-diet variation was greater than within-diet variation (**Figures 1G, H**), with xylan-fed communities producing distinct communities compared to the other synthetic diets. Ordination analyses using non-metric multidimensional scaling (NMDS) and PERMANOVA analysis showed that samples clustered based on diet composition in both weighted (**Figure 1B**; PERMANOVA: R^2^ = 0.42; p<0.001) and unweighted (**Figure 1C**; PERMANOVA: R^2^ = 0.329; p<0.001) Bray–Curtis metrics, with especially clear separation of the xylan-based diet from other synthetic diets. Removing the xylan-fed samples from the diversity calculations did not eliminate diet-based clustering for weighted (**Supplementary Figure S1A**; PERMANOVA: R^2^ = 0.343; p<0.001) or unweighted (**Supplementary Figure S1B**; PERMANOVA: R^2^ = 0.247; p<0.001) measures, suggesting that each carbohydrate source enriched for a unique community composition.

### 3.2 Diet-characteristic taxa enriched by polysaccharide source

We used DESeq2 to identify 76 microbes that exhibited significantly higher abundance in a single synthetic diet across pairwise comparisons against all other treatments, which we termed “diet-characteristic taxa” (**Supplementary Figure S2**) (Love et al., 2014). Diet-characteristic ASVs were primarily assigned to Firmicutes (n=48) and Bacteroidota (n=20); other phyla with diet-responsive taxa include Fusobacteriota, Deferribacterota, Desulfobacterota, Fibrobacterota, and Spirochaetota. We found that the chitin and methylcellulose diets were not associated with any diet-characteristic taxa by this definition, while the diets made with xylan, MCC, starch, and pectin enriched for 45, 10, 13, and 8 ASVs respectively.

### 3.3 Cohort effects on diet-driven differences in gut microbiome composition

**Figure 2:**
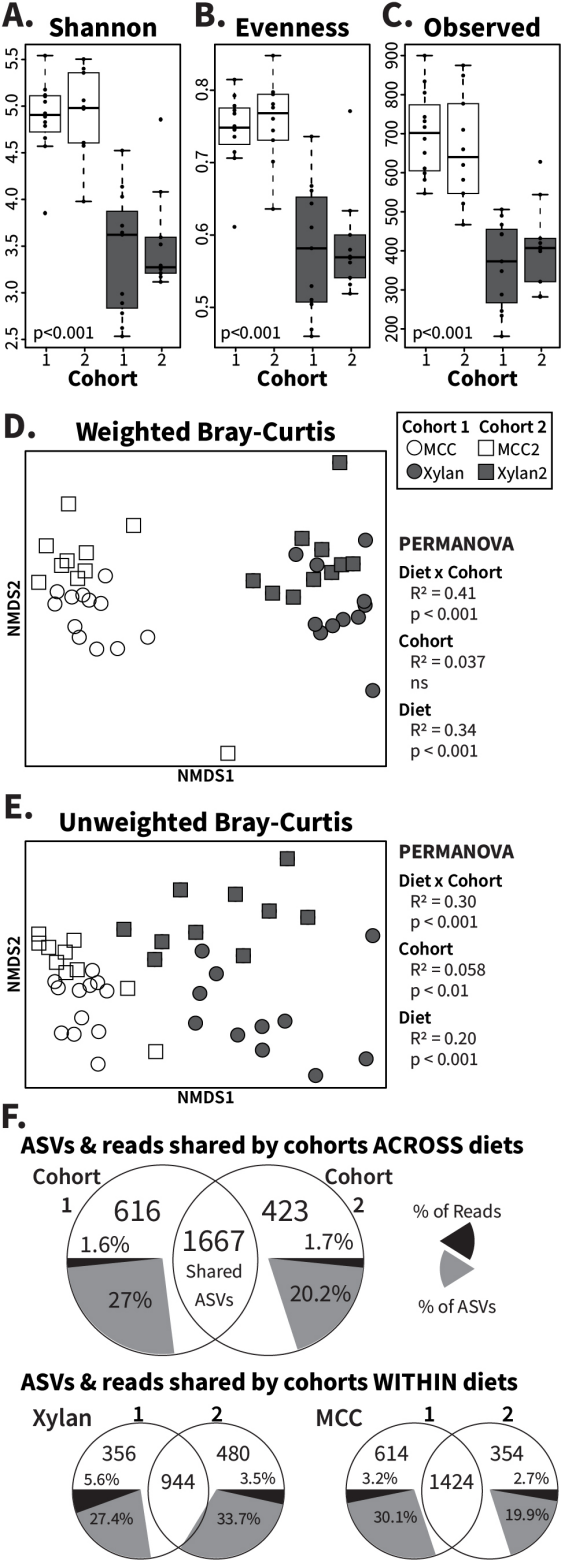
Analysis of cohort effects on gut microbiome responses to MCC and xylan diets. Xylan and MCC-fed samples from replicate experiments were rarefied to 9685 ASVs for alpha and beta diversity assessment. Boxplots show **(A)** the Shannon index, **(B)** Pielou’s evenness, and **(C)** the number of observed ASVs with Kruskal–Wallis p-values calculated across all individual groups. PERMANOVA was used to calculate R^2^ and p-values for diet (“MCC” and “Xylan”), cohort (“Cohort 1” and “Cohort 2”), and diet x cohort for NMDS ordinations of **(D)** weighted and **(E)** unweighted Bray–Curtis dissimilarity, with one point plotted per insect. The last panel **(F)** contains Venn diagrams of shared and unique ASVs between cohorts for both diets together as well as separately, constructed using rarefied count tables collapsed by diet and/or cohort. Grey pie slices represent the percentage of ASVs observed that are cohort-unique, while black pie slices represent the percentage of sequence reads assigned to the indicated unique ASVs. ns, no significance.

To confirm that the diet-associated gut community differences we observed are genuine rather than artifacts of natural variation in the insect colony (“cohort effects”), we prepared fresh MCC- and xylan-based diets and repeated the 2-week diet experiment with a new cohort of adult cockroaches. These diets were selected for follow-up experiments due to both their contrasting molecular structure and the dissimilarity they generated in the Bray–Curtis analyses (**Figures 1B, C**). Data from the first and second experiments exhibited similar alpha diversity measurements (**Figures 2A–C**), with the repeated cohorts maintaining the significant shifts in alpha diversity (p<0.001) observed in the initial experiment between these two diets while showing no difference between the same-diet cohorts. Beta diversity analysis showed that samples clustered by both cohort and diet (**Figures 2D, E**). Diet had large effects on both weighted (PERMANOVA: R^2^ = 0.34; p<0.001) and unweighted (PERMANOVA: R^2^ = 0.20; p<0.001) Bray–Curtis dissimilarity, while the cohort explained minimal effect sizes of 3.7% and 5.8% for the weighted and unweighted measures respectively, with only unweighted reaching significance (p<0.01).

Because unweighted Bray–Curtis calculations only consider whether an ASV is present or not in an individual community, we hypothesized that the difference between the cohorts was driven by low-abundance microbes that had lesser impact in the calculation of abundance-weighted beta diversity. Using Venn diagrams (**Figure 2F**), we confirmed that most of the ASVs recovered were in fact shared across cohorts. While 616 ASVs (27%) were unique to cohort 1 and 423 ASVs (20.2%) unique to cohort 2 (**Figure 2F**, grey pie slices), these ASVs represented only a small fraction of the total sequences (**Figure 2F**, black pie slices) obtained from each cohort. Further, 66.7% (cohort 1) and 61% (cohort 2) of these ‘cohort-specific’ taxa appeared in only one sample (**Supplementary Figure S3**), indicating that most of the differences in composition due to the time between the studies stemmed from transient, rare taxa. Separating the diets for these comparisons confirmed the overall findings, with rare taxa contributing few sequencing reads despite comprising 19.7%-33.7% of the unique ASVs (**Figure 2F**). Altogether, these results show that synthetic diets reproducibly alter the gut microbiome composition in cockroaches.

### 3.4 Testing the impact of alternative diet formulations

While fiber is the primary component of undigested material that reaches hindgut microbiota, other macro- and micronutrients are known to influence gut community structure in non-cockroach host systems (Zhu et al., 2015; Zhao et al., 2017; Akimbekov et al., 2020). To confirm the role of polysaccharides as key modulators of the gut community, we leveraged our synthetic diets to test the impacts of xylan and MCC-based diets deficient in protein or micronutrients. For protein-deficient diets (**Table 1**), casein and peptone were replaced by mass with either xylan or MCC, while in vitamin-deficient diets, both the vitamin mixture and cholesterol were replaced with additional polysaccharides. We also created simple sugar versions of these two diets to test whether replacing long-chain fibers with their component backbone sugars results in different gut communities: xylose for comparison with xylan, and cellobiose and glucose for comparison with MCC.

**Figure 3:**
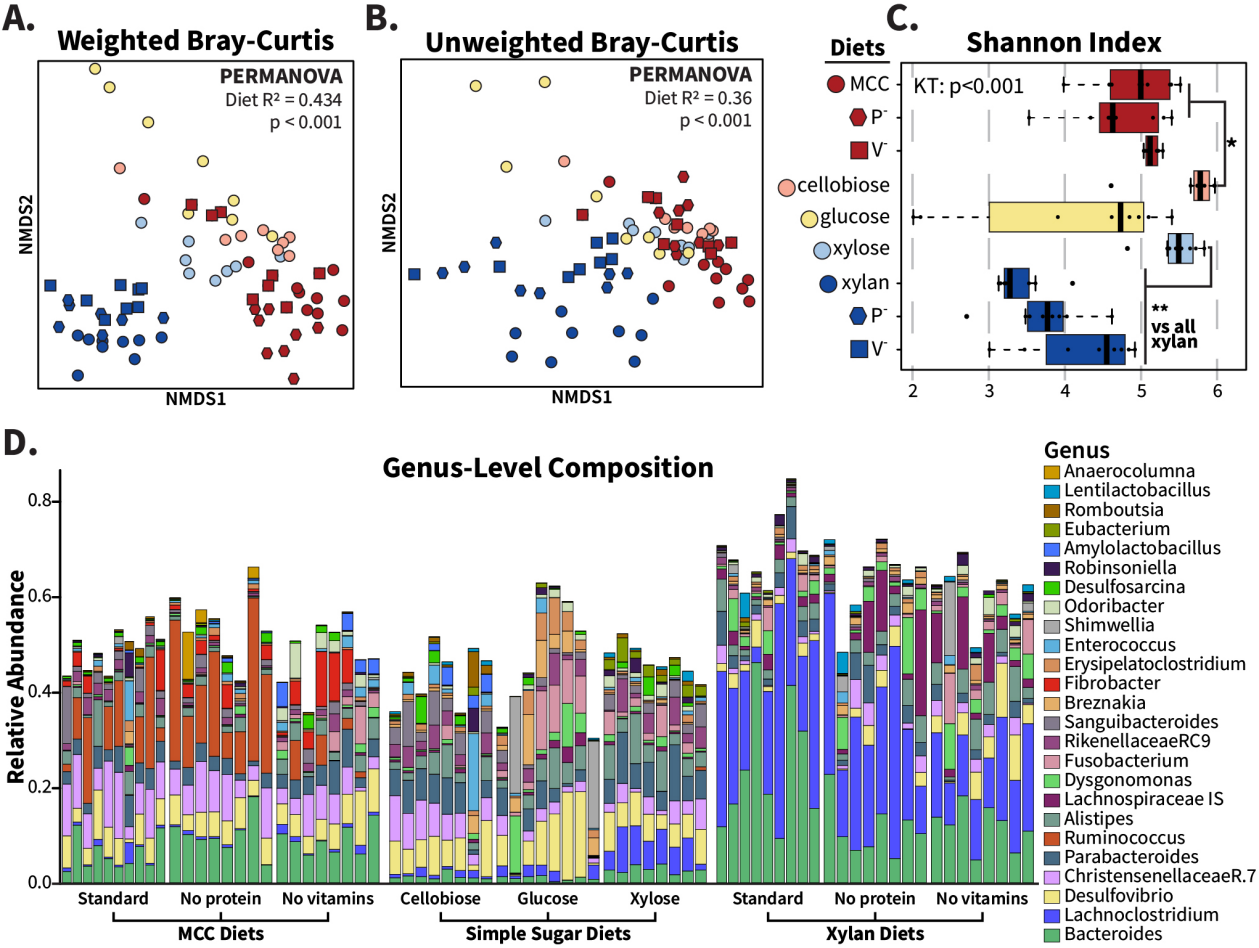
Fiber source, not protein, vitamins, or sugar composition, determines community structure from xylan and MCC-based synthetic diets. Deficient and simple sugar variations of MCC and xylan synthetic diets were fed to adult cockroaches for two weeks, and hindgut community compositions were compared with replicated xylan and MCC samples. For these analyses, samples were rarefied to 12274 reads and plotted with each point representing one individual. NMDS ordinations were made for **(A)** weighted and **(B)** unweighted Bray–Curtis dissimilarity, and PERMANOVA used to calculate R^2^ and p-values with “diet” as the grouping factor. Alpha diversity is displayed via **(C)** the Shannon index with the Wilcoxon rank-sum test used for pairwise comparisons. The relative abundance of abundant genera found in the MCC- and xylan-based diets are visualized in **(D)**. * =p <0.05; ** =p<0.01.

In unweighted Bray–Curtis analyses, which consider only the presence/absence of ASVs (**Figure 3B**), communities from all MCC-fed insects overlapped with those of the sugar diets while retaining separation from xylan-fed samples (PERMANOVA: R^2^ = 0.36; p<0.001) suggesting that MCC and the sugar diets supported a shared set of microbiota that were absent in the xylan-fed insects. When abundance of ASVs is accounted for via weighted Bray–Curtis ordination (**Figure 3A**), the sugar-fed communities formed their own distinct cluster, while both xylan-fed and MCC-fed samples clustered by polysaccharide regardless of vitamin or protein content (PERMANOVA: R^2^ = 0.434; p<0.001). The alpha diversity profiles of the deficient diets matched the standard MCC or xylan diets as well, as measured by the Shannon index (**Figure 3C**), evenness (**Supplementary Figure S4A**), and number of observed ASVs (**Supplementary Figure S4B**). The cellobiose communities displayed slightly higher Shannon index values than the standard and protein-deficient MCC communities, while the xylose-fed insects possessed noticeably more even and diverse communities than the xylan diets. At the genus level, (**Figure 3D**) the community composition of the insects fed diets containing the same polysaccharide resembled each other, while sugar-fed microbiota reflected each other more than the polysaccharide they were derived from. Despite the xylose-fed samples clustering with the other sugar diets, the pentose diet did enrich for an abundant *Lachnoclostridium* ASV (**Supplementary Figure S5**) that is heavily associated with xylan, while MCC-associated taxa such as *Fibrobacter* and *Ruminococcus* remained at low levels in the glucose and cellobiose diets. Overall, these results suggest that the communities observed in the fiber diets are driven by the long-chain structures of the polysaccharides rather than their component sugars.

### 3.5 Comparison with whole-food diets

The different microbial communities triggered by our synthetic diets were unexpected given that previous experiments examining the impact of whole-food diets with strongly differing macronutrient profiles did not produce substantially different gut microbiome compositions (Tinker and Ottesen, 2016). Therefore, we compared the samples from this current study (“synthetic” diet type) to samples from the previous study (“whole food” diet type) of cockroaches fed butter, tuna, honey, white flour, or whole wheat flour (Tinker and Ottesen, 2016). Both studies fed diet treatments *ad libitum* to groups of adult mixed-sex American cockroaches for 2 weeks, following the experimental setup described in Methods section 3.3.

**Figure 4:**
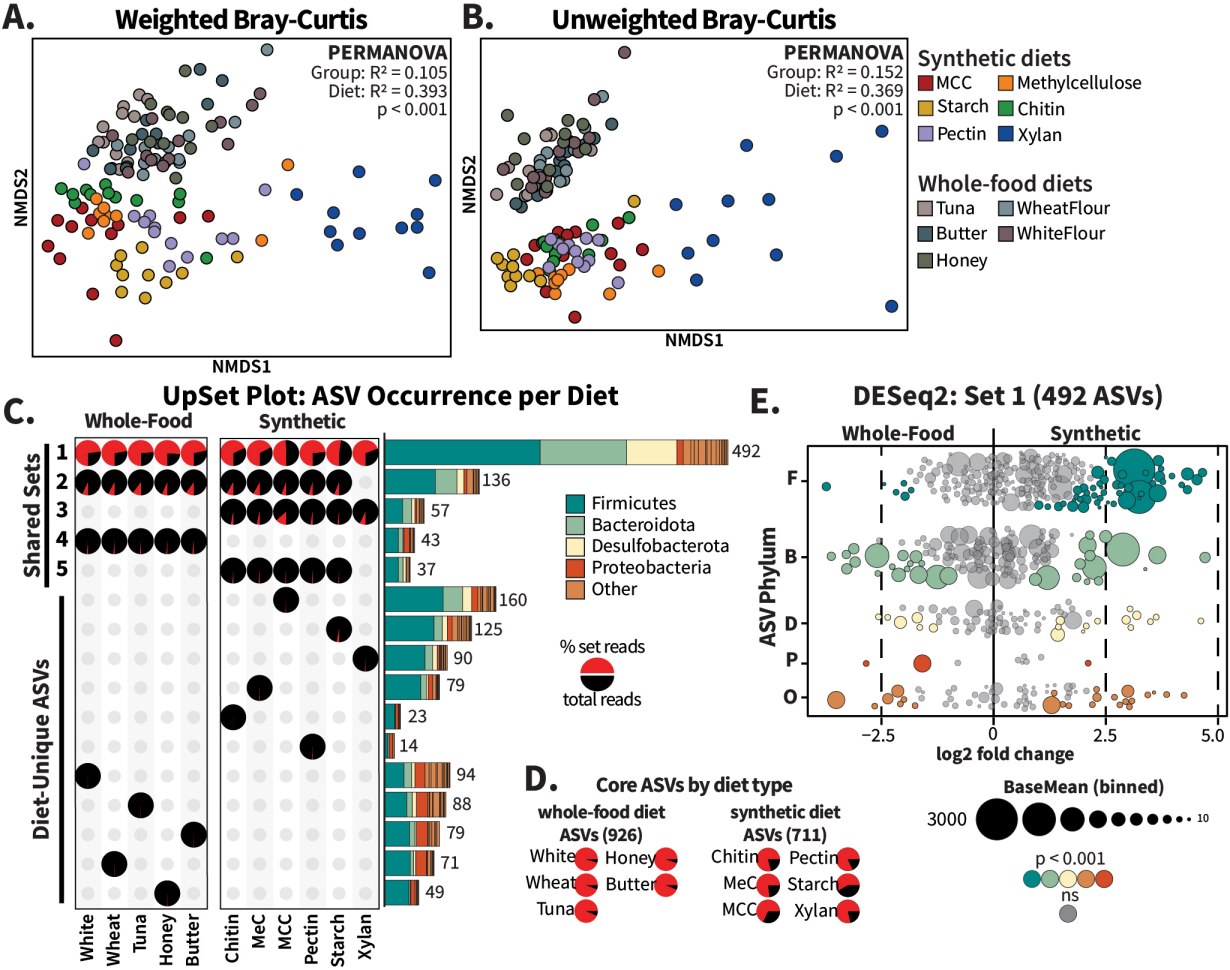
Whole-food diets share more ASVs than synthetic diets. Raw sequence data from Tinker and Ottesen (2016) for cockroaches fed tuna, butter, honey, wheat flour, and white flour (“Whole Food Diets”) were reprocessed using the methods in this experiment to generate comparable ASVs. For beta diversity comparison, all samples were rarefied to 7,924 ASVs and NMDS ordinations, with one point per insect, were generated for **(A)** weighted and **(B)** unweighted Bray–Curtis distances; R^2^ and p-values for diet type comparisons were calculated using PERMANOVA. For **(C)** UpSet plot analysis, the five largest intersections (Sets 1-5) and diet-unique sets are displayed. Pie charts represent the percentage of reads within a diet that originated from each set (red slices), and the bar charts are colored to display the phylum-level distribution of ASVs assigned to each set. Read abundance of core ASVs, or those present in all synthetic or whole-food diets regardless of presence in the other diet type, are visualized in **(D)** pie charts per diet. For the MA plot in panel **(E)**, raw sequence count tables for the ASVs identified as “Set 1” were analyzed using DESeq2 with diet type as the design factor. The ASV circles are scaled according to baseMean size and colored by phylum.

We found that the gut microbiome samples from cockroaches fed synthetic diets exhibited higher ASV richness (p<0.01) but lower evenness (p<0.001) and Shannon index values (p<0.01) than those from insects fed whole foods (**Supplementary Figures S6A-C**). Synthetic and whole-food diets produced distinct diet type clusters in the NMDS ordinations (**Figures 4A, B**) for weighted (PERMANOVA: R^2^ = 0.105; p<0.001) and unweighted (PERMANOVA: R^2^ = 0.152; p<0.001) Bray–Curtis dissimilarities. When we analyzed the samples by diet, we found that diet explained more variation in the NMDS ordination than diet type as interpreted from PERMANOVA R^2^ values (weighted: R^2^ = 0.393; unweighted: R^2^ = 0.369).

Beta dispersion analysis of variation within diet types showed that, together, the gut microbiota of cockroaches fed synthetic diets was more variable than that observed among whole-food-fed cockroaches (**Supplementary Figures S7A, B**; Tukey’s HSD: p<0.001). However, when diets were analyzed individually, they were equally dispersed in weighted Bray–Curtis dissimilarity (**Supplementary Figure S7A**) but not unweighted measures (**Supplementary Figure S7B**; ANOVA: p<0.001). The xylan-fed cockroaches exhibited significantly greater within-group variability than the ten other diets (Tukey’s HSD range: p = 0.037 – 3.17e^-06^), with no significant differences observed in the pairwise comparisons of all other diets. When synthetic diets were compared to whole-food diets without including xylan-fed samples, we observed no significant differences in unweighted beta dispersion (**Supplementary Figure S7D**) or Shannon index values (**Supplementary Figure S6B**, red boxes). However, significant differences remained in weighted Bray–Curtis dispersion (**Supplementary Figure S7C**), richness and evenness (**Supplementary Figures S6A, S6C**), and in both weighted and unweighted Bray–Curtis ordination analysis (**Supplementary Figures S8A, B**), highlighting that the altered microbiomes produced by the synthetic diet type were not solely due to biases produced by xylan-fed samples.

To verify that the inclusion of whole-food dietary components alone was not sufficient to eliminate fiber-dependent gut microbiome configurations, we tested the impact of diets mimicking our synthetic diets but with the purified protein components replaced with canned tuna. These diets induced community compositions similar to those observed in polysaccharide-matched diets containing purified proteins rather than supporting protein-associated communities (**Supplementary Figure S9**). Xylan-containing diets generally produced communities with lower alpha diversity scores (**Supplementary Figures S9D-F**) and clustered away from the MCC-containing diets and dog food-fed insects we included as controls in weighted (**Supplementary Figure S9B**; PERMANOVA: R^2^ = 0.395; p<0.001) and unweighted (**Supplementary Figure S9C**; PERMANOVA: R^2^ = 0.392; p<0.001) analyses. Despite the discordant structural complexities between tuna fish and purified casein/peptone amino acids, the protein portion of the synthetic diets exerted less influence than the polysaccharide source.

### 3.6 Core taxa differences between synthetic and whole-food diets

Given these strong differences in community structure, we utilized the R package UpSetR to determine how the ASVs in the different diet types overlap (Conway et al., 2017). UpSet plots are akin to Venn diagrams, considering only the presence/absence of an ASV. Rarefied count tables were aggregated by diet and ASVs were marked as either present or absent per diet. ASVs present in the same subset of diets were grouped into “Sets”, with the phylum-level composition per set depicted as stacked bar charts labeled with the number of included ASVs (**Figure 4C**). We supplemented the UpSet plot with pie charts illustrating the relative abundance (calculated as the fraction of total reads recovered from the collapsed treatment group) of reads assigned to ASVs within each set (**Figure 4A**), in addition to pie charts representing the “core” ASVs present in all whole-food or all synthetic diets, regardless of presence in the other dietary group (**Figure 4D**). For simplicity’s sake, **Figure 4** and **Supplementary File 1** show only the five largest intersecting sets as well as all single-diet sets; additional sets are presented in **Supplementary Figure S10**.

A total of 492 ASVs (“Set 1”) were shared across all diet treatments (**Figure 4C**). These ASVs made up over half of the sequences recovered for all diets except the MCC diet, for which they represented 49% of sequences (**Figure 4C**, pie charts). Only 43 ASVs (“Set 4”) were exclusive to the whole-food diets, contributing between 0.9% and 1.65% of the reads in these diet sets. The 57 ASVs (“Set 3”) identified as exclusive to the synthetic diets made up 1.6%-3.4% of the reads recovered from the starch-, pectin-, chitin-, and methylcellulose-fed cockroaches, and 7% of the xylan-fed and 13% of MCC-fed cockroaches. Together, these results indicate that the synthetic diets did not eliminate the core taxa present in the guts of cockroaches fed whole foods, nor did they result in hindgut colonization by a large new set of microbial taxa.

Similarly, individual synthetic diets were not associated with hindgut colonization by large groups of unique microbes. In general, taxa that were unique to individual synthetic or whole-food diets represented a very small proportion of sequences recovered (**Figure 4C**). ASVs unique to the MCC diet formed the second largest set overall, with 160 diet-unique taxa, yet they only represented 0.4% of total recovered sequences (**Figure 4C**). A xylan-based diet, which repeatedly produced the largest community dissimilarities (**Figures 4A, B**; **Supplementary Figure S7**), was associated with 90 diet-specific taxa comprising only 0.63% of reads. Interestingly, our analysis revealed that a xylan-based diet did result in the loss of 136 taxa that were present in all other diets in abundances ranging from 4.78%-10.14% (“Set 2” in **Figure 4C**). However, other sets that excluded individual diets were substantially smaller (**Supplementary Figure S10A**) suggesting that this was not a common mechanism underlying the diet-driven differences in gut microbiome composition. Instead, synthetic diet-driven differences in gut microbiome composition were primarily associated with high relative abundance of individual taxa that were consistently present in the cockroach gut regardless of dietary treatment.

To follow up on these observations, we used DESeq2 to assess the enrichment of individual ASVs between synthetic- vs whole-food-fed cockroaches. Of the 492 ASVs found, often at high abundances, in all 11 diets, 95 ASVs were significantly enriched in cockroaches fed synthetic diets while 38 ASVs were enriched in cockroaches fed whole-food diets (**Figure 4E**; padj < 0.001). The magnitude of these ASV-level differences across diet type were modest (log fold change <5), consistent with the high proportion of reads recovered in all diets that belonged to Set 1 (**Figure 4C**). Bacteroidota, Desulfobacterota, and other phyla showed similar enrichment distributions to each other in terms of both the number of ASVs and abundance within samples, but the 49 Firmicutes enriched in the synthetic diets included far higher individual abundances than the six enriched in the whole-food diets.

We also examined the enrichment of ASVs that fell outside of Set 1 but were both somewhat abundant (baseMean > 1) and present in at least five samples (**Supplementary Figure S11**). These 1,270 ASVs generally had smaller abundances than the members of Set 1 (**Figure 4E**), but greater log-fold changes between diet types. The ASVs *Ruminococcus_NA* and *Fusobacterium ulcerans* were exceptions, being both highly abundant (baseMean = 1020 and 651, respectively) and unique to the synthetic diets. In contrast, the *Christensenellaceae* ASV (R7_NA.68), unique to whole-food diets, had a high log-fold change, but a baseMean of only 4.29. Apart from a few highly abundant ASVs in the synthetic diets, most of the abundant, diet-enriched taxa were shared by all diets, supporting that change is driven by common gut bacteria restructuring the community rather than interloping bacteria disrupting the community.

### 3.7 Differential diet-based fluctuations of abundant Firmicutes and Bacteroidota ASVs

**Figure 5:**
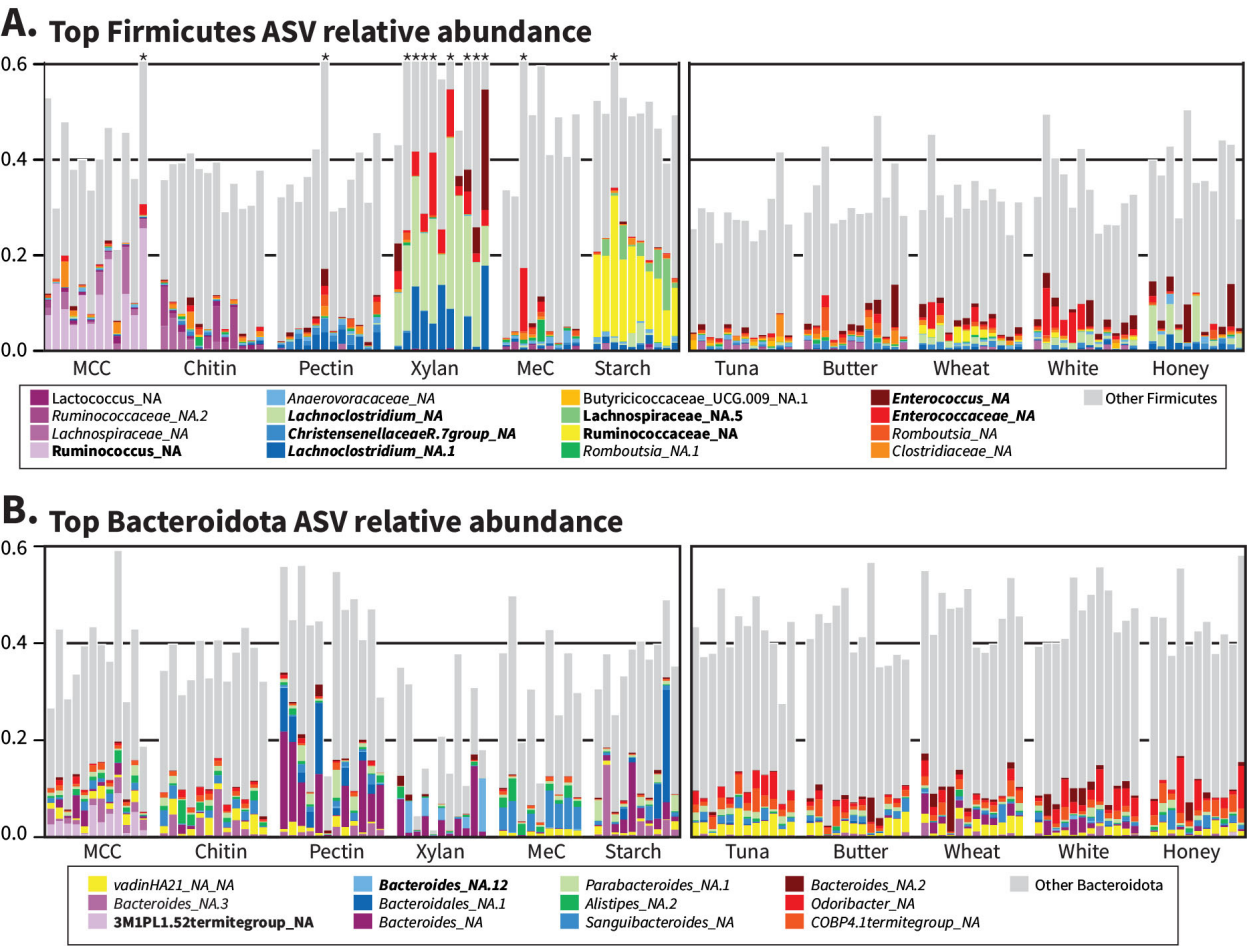
Individual ASVs explain large differences between synthetic but not whole-food diets. Variance-stabilized count data from DESeq2 were used to determine the top two ASVs for every diet belonging to **(A)** Firmicutes and **(B)** Bacteroidota, and the relative abundances of ASVs belonging to the combined ‘top ASV’ set were plotted for all individual samples. Grey bars include all Firmicutes or Bacteroidota not named in the key. * indicates “Other Firmicutes” that extend beyond 60% relative abundance; please refer to **Figure 1A** for full values. Names in bold indicate diet-characteristic ASVs from **Supplementary Figure S2**, and italics indicate Set 1 ASVs from **Figure 1C**.

To explore the impact of diet on microbial taxa associated with fiber degradation, we evaluated the dietary responses of abundant taxa within the Firmicutes and Bacteroidota. For this analysis, we selected the two most abundant representatives of each of these phyla from each diet and examined their abundance across all diets (**Figure 5**). We identified 16 Firmicutes and 11 Bacteroidota as most or second-most abundant in at least one diet.

The most abundant taxa from both Firmicutes and Bacteroidota represented a small fraction of reads across the whole-food diets, consistent with the higher Shannon diversity and evenness of the gut microbiome for whole-food-fed cockroaches (**Supplementary Figures S6B, C**). In contrast, the synthetic diets produced strong ‘blooms’ of individual ASVs, particularly among the Firmicutes (**Figure 5A**). Several abundant Firmicutes were both present in Set 1 (**Figure 1C**) and enriched in individual synthetic diets (**Supplementary Figure S2**), namely *Lachnoclostridium_NA* (xylan), *Lachnoclostridium_NA.1* (xylan), *Enterococcus_NA* (xylan), *Enterococcaceae_NA* (xylan), and *ChristensenellaceaeR7_NA* (pectin). In contrast, the Firmicutes identified as enriched in the MCC and starch diets were not typically found across all diets: *Ruminococcus_NA* (MCC), *Lachnospiraceae_NA.5* (starch), and *Ruminococcaceae_NA* (starch). Among the Bacteroidota (**Figure 5B**), we observed greater overlap in the most abundant taxa present in each diet group, with all but one (*3M1PL1.52termite_NA*) of the abundant Bacteroidota ASVs belonging to Set 1, and only two classified as diet-characteristic: *3M1PL1.52termite_NA* (MCC) and *Bacteroides_NA.12* (pectin).

### 3.8 Analysis of microbial co-correlation networks

**Figure 6:**
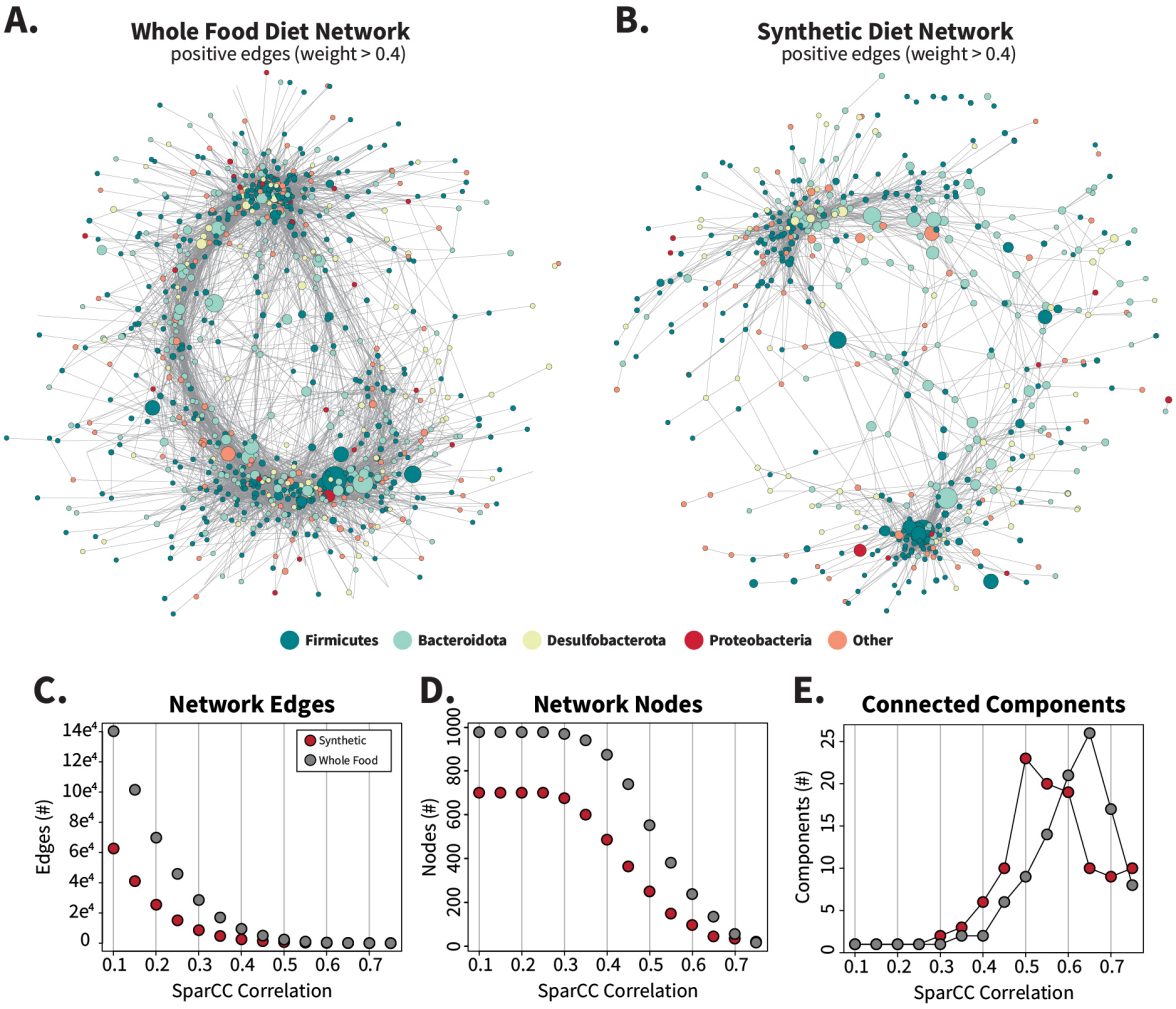
Synthetic diet correlation networks are smaller and less interconnected than whole-food diet correlation networks. Networks were calculated by SparCC from filtered count tables (ASVs present in > 25% of samples per diet set) for synthetic and whole-food diets separately to create two distinct networks containing 976 (whole food) or 700 (synthetic) nodes. Networks were imported into Cytoscape and edges with absolute values < 0.4 were removed to generate panels **(A, B)**. Negative edges were included during initial layout generation with the edge-weighted spring-embedded layout method and are displayed in **Supplementary Figure S12**. The number of nodes, edges, and connected components that remain when the networks are filtered by increasing correlation values are charted in **(C–E)** respectively.

We constructed co-correlation networks with SparCC to examine the community structure underlying synthetic and whole-food microbiome data sets (Friedman and Alm, 2012). To filter out noise and reduce spurious correlations, the datasets were filtered to include ASVs present in at least 25% of the samples for each diet type, resulting in 976 ASVs for whole-food diets and 700 ASVs for synthetic diets. The networks were further pruned to contain edge weights with absolute values larger than 0.4, removing 75 and 168 ASVs from the whole-food and synthetic networks respectively. Both positive and negative edges were retained for network layout formation (**Supplementary Figure S12**), but for analysis, only positive edges were considered (**Figure 6**). After negative edge removal, the whole-food network contained 875 ASVs with 9,515 edges forming two connected components (**Figure 6A**), while the synthetic diet network contained 497 ASVs with 2536 edges that formed six connected components (**Figure 6B**). Networks at SparCC correlation levels of 0.5, 0.6, and 0.7 are included in **Supplementary Figure S13**.

Gut microbiota from the whole-food-fed cockroaches formed an extensive and dense interaction network (**Figure 6A**), with higher edge counts (**Figure 6C**), node counts (**Figure 6D**), node degree (**Supplementary Figure S14A**), network strength (**Supplementary Figure S14B**), and betweenness scores (**Supplementary Figures S14C, D**) than those fed synthetic diets at most levels of filtering based off SparCC correlation values. High-degree nodes, or ASVs with large numbers of neighbors, were present throughout the whole-food network structure, while in the synthetic diets, two primary clusters of ASVs appeared with fewer connecting ASVS. When a range of inclusion cutoffs was considered, the synthetic diet network degraded more quickly than the whole -food network into separate connected components (**Figure 6E**; **Supplementary Figure S13**) displaying greater fragility and a tendency to fragment. These overall network structures suggest that the synthetic diets disrupted the stability of the cockroach microbiome.

## 4 Discussion

The core finding of this work is that synthetic diets featuring a single purified complex polysaccharide source induced distinctive and fiber-dependent hindgut microbiome compositions in omnivorous cockroaches. Three of our polysaccharides are abundant structural components in the plant cell wall: cellulose (β-1,4-linked glucose), xylan (β-1,4-linked xylose), and pectin (α-linked galacturonic acid and/or rhamnose). In a typical plant cell, chains of cellulose interface with hemicellulose (xylan or xyloglucan: β-1,4-linked glucose with α-1,6-linked xylose residues) to form a rigid scaffold interspersed with a pectic polysaccharide gel matrix, with these components fortified via hydrogen bonding to create the stable cell wall (Waldron et al., 2003). In contrast to cell wall polysaccharides, which are expected to require bacterial fermentation for efficient degradation, the polysaccharides starch (amylose: straight chain of α-1,4-linked glucose; amylopectin: branched chain of α-1,4 and α-1,6-linked glucose) and chitin (*N-*acetyl-β-D-glucosamine chain) are more easily digestible by the cockroach. Starch can be digested by both salivary and midgut-derived α-amylases to provide energy, although the efficiency of these enzymes depends on the amylose/amylopectin makeup of the starch granules. Chitin is a key component of the insect cuticle that is recycled through consumption of the exuviae after molting as well as consumed during cannibalism (Merzendorfer and Zimoch, 2003), but the level of chitinase activity in the midgut itself is poorly quantified. Methylcellulose is the only compound tested in this study that is not a naturally occurring polysaccharide; rather, it is a synthetically modified cellulose with an average of 1.8 hydroxyl groups per glucose residue replaced by methoxide. It was selected as a water-soluble cellulose derivative that is also commonly used as an “inert” emulsifier and can act as a laxative.

The key changes observed in this study include alterations in the abundance of multiple organisms, but especially bacteria from the phyla Bacteroidota and Firmicutes (**Figure 5**). Members of the Bacteroidota are thought to be key fiber-degrading organisms in cockroaches, supported by the many carbohydrate-active enzymes (CAZymes) encoded both within and independently of polysaccharide utilization loci (PULs) in multiple isolates’ genomes (Vera-Ponce de León et al., 2020; Dukes et al., 2023). The high gene density of CAZymes likely allows Bacteroidota members to efficiently ferment diverse fibers without exclusively relying on a single source (Vera-Ponce de León et al., 2020; Dukes et al., 2023). While Firmicutes comprise a large proportion of the bacteria in the cockroach gut, their roles in fiber degradation remain elusive due in part to their extensive genomic diversity (Dukes et al., 2023), and their sparse roles in polysaccharide fermentation in the cockroach’s termite relatives (Salgado et al., 2024). In ruminant research, the Firmicutes species *Ruminococcus flavefaciens* and *Ruminococcus albus* have been extensively studied for their powerful cellulolytic capabilities (Israeli-Ruimy et al., 2017; Drula et al., 2022). However, in cockroaches and in humans, fiber-degrading Firmicutes are characterized by lower gene densities of CAZymes than Bacteroidota, suggesting they function as secondary polysaccharide fermenters that scavenge materials released from cell matrices by generalist Bacteroidota (El Kaoutari et al., 2013; Dukes et al., 2023; Sun et al., 2023).

Interestingly, of the six polysaccharides used in this study, only the four plant-derived polysaccharides (MCC, xylan, starch, and pectin) were associated with diet-characteristic ASVs (**Supplementary Figure S2**). The chitin and methylcellulose diets, in contrast, produced communities with the highest alpha diversity values (**Figures 1D, E**) and clustered together in beta diversity analyses (**Figures 1B, C**; **Supplementary Figure S1**) closest to the whole-food diets (**Figure 4A**). Chitin is present in the cockroach hindgut even in the absence of a dietary source due to the continuous shedding of the PM from the midgut, which may explain why no unique organisms were selected for in this diet condition. Methylcellulose, on the other hand, may resemble fiber starvation from the perspective of the gut bacteria, which has previously been shown not to alter the gut microbiota (Tinker and Ottesen, 2016). Methylcellulose has been found to reduce adhesion and inhibit cellulase (but not cellobiase) activity in the rumen bacteria *R. albus*, *R. flavefaciens*, and *Fibrobacter succinogenes* (Rasmussen et al., 1988; Sung et al., 2013). The extent to which this polymer interacts with other fibrolytic systems, such as those in Bacteroidota, is unclear, but our results suggest that it does not select for a unique set of gut microbes.

Among the polysaccharides tested, the hemicellulose xylan induced the largest shifts in alpha diversity, inter-individual variability, and overall community composition (**Figure 1**). Xylans are abundant heteropolysaccharides that vary in branch complexity according to the source they are derived from, ultimately influencing their digestibility for hindgut and rumen microbiota (Dehority, 1967; Coen and Dehority, 1970; Hespell and Cotta, 1995). The xylan in this study is derived from corn cob and contains residues of galactose, arabinose, and glucuronic acid with low levels of acetylation (Gírio et al., 2010). Research performed *in vitro* investigating the xylan degradation ability of gut microbiota mainly focuses on *Bacteroides* (Dodd et al., 2011; Zhang et al., 2014; Centanni et al., 2017; Kmezik et al., 2021; Pereira et al., 2021), although clostridial organisms such as *Roseburia intestinalis* and the rumen bacterium *Butyrivibrio fibrisolvens* have also been identified as key butyrate-producing xylan fermenters (Hespell and Cotta, 1995; Mohand-Oussaid et al., 1999; Leth et al., 2018; Hershko Rimon et al., 2021). Interestingly, we found that in cockroaches, xylan-based diets decreased the relative abundance of Bacteroidota, while increasing Firmicutes (**Figure 1A**). Concurrent with this enrichment of specific taxa, xylan decreased the overall diversity of the gut community (**Figure 1**); in contrast, feeding the monomer xylose to cockroaches retained high community diversity while still selecting for a *Lachnoclostridium* ASV that was substantially enriched in the xylan diet (**Figure 3**; **Supplementary Figure S5**).

Direct comparison of these results with other *in vivo* dietary studies is difficult, as livestock studies utilizing it as a dietary additive frequently produced harmful effects such as lower ileal digestibility of essential amino acids in pigs and a proliferation of pathogens in broilers [reviewed in (Baker et al., 2021)]. However, an abundance of research has been performed analyzing the effects of xylan-containing whole foods and its derivatives on gut microbiota (Broekaert et al., 2011; Jefferson and Adolphus, 2019; Jana et al., 2021; Smith and Melrose, 2022), with notable enrichment of *Lachnospiraceae* species on both xylo-oligosaccharides (XOS) and xylan-containing whole foods (Chung et al., 2019; Liu et al., 2020a; Berger et al., 2021; Zhou et al., 2021). Co-culture assays performed using commensal *Bacteroides* and *R. intestinalis* identified different transporter affinities for xylan degradation products based on XOS size (Leth et al., 2018), while studies using both purified hemicellulose and intact forage found that *R. flavefaciens* effectively converted some xylans to acid-soluble forms but required a co-culture with *B. fibrisolvens* to grow (Dehority, 1967; Coen and Dehority, 1970). The purified xylan used in this work seems to select for Firmicutes with specialized xylan degradation machinery. This advantage was minimized in the xylose diet, allowing other microbiota to grow concurrent with the enriched *Lachnoclostridium* ASV.

While xylan-based diets induced the largest differences in hindgut community composition, samples clustered by dietary treatment even when the xylan treatment group was excluded (**Supplementary Figure S1**). These results stand in stark contrast to previous works from multiple investigators, who found minimal to no differences in hindgut microbial community composition in response to diet alterations (Schauer et al., 2014; Tinker and Ottesen, 2016; Lampert et al., 2019). A commonality between these experiments is that the investigators utilized whole-food diets or animal feeds containing processed complex plant material such as milled bran or soymeal. On the other hand, other investigators have observed a substantial influence of diet on the gut microbiome composition (Bertino-Grimaldi et al., 2013; Pérez-Cobas et al., 2015; Zhu et al., 2022). These experiments all utilized synthetic diets that contained purified, lab-generated carbohydrate and protein sources without the undefined cell matrix components that are retained in “whole food” or animal feed diets. For example, in experiments using *B. germanica*, Pérez-Cobas et al. (2015) prepared synthetic diets with a dextrin and micronutrient base amended with either 50% cellulose or 50% casein while Zhu et al. (2022) used diets composed of a cellulose and micronutrient base supplemented with 40% mass purified starch, casein, or sesame oil. In *P. americana*, Bertino-Grimaldi et al. (2013) utilized purified cellulose to compare with sugarcane bagasse, a complex dietary substrate. Given these conclusions and the findings described in our study, it appears that synthetic diets combined with the selective ability of purified fibers can produce marked differences in the cockroach gut community, although the paucity of cockroach studies with standardized dietary methods limits the strength of these conclusions. Future research is required to conclusively place weight on the purified nature of these diets in the context of cockroach gut microbiota. In addition, we note that the lack of compositional differences does not preclude functional differences resulting from changes in microbial activity, as reported in Schauer et al. (2014) and DePoy et al. (2024).

Looking beyond cockroach models, there appears to be a similar influence of synthetic diets containing highly purified components on gut microbial composition in numerous insect and mammalian studies. Termites, a close relative to cockroaches, responded to single carbohydrate source diets with larger alterations in gut community composition than termites fed mixed-carbohydrate diets (Miyata et al., 2007). Other insect model systems produced similar results, such as in silkworms (Dong et al., 2018), ladybugs (Xie et al., 2024), waxworms (Gohl et al., 2022), and honeybees (Powell et al., 2023). Among mammals, dogs provided a purified diet also exhibited reduced alpha diversity compared to those fed a complex diet (Allaway et al., 2020), wild-caught mice transitioned from natural diets to laboratory diets lost large portions of native gut microbes (Martínez-Mota et al., 2020), and humans given meal replacement shakes showed a loss of biodiversity in their microbiome compositions (Gurry et al., 2018).

Comparison of our dataset with data recovered in previous whole-food-based dietary experiments suggests that synthetic diets altered the gut community by inducing the overgrowth of microbes already present in the cockroach gut microbiome (**Figure 4** **),** a similar outcome to *in vitro* enrichment one may perform on selective media. Taxa that were unique to individual diets represented <1% of sequence reads in all diets but starch, of which they made up 2.84% (**Figure 4**). In contrast, 15 out of 20 highly abundant Firmicutes and Bacteroidota associated with one or more diets were shared across all diet types, while the remaining 5 were found sporadically in other diets (**Figure 5**). The alterations we observed were especially associated with the fibers themselves rather than the other dietary components. Experiments leveraging the xylan and MCC diets without amino acids (**Figure 3**) or with tuna (**Supplementary Figure S9**), a complex food, substituted for the casein/peptone mixture of our standard diet configuration largely did not differ from the polysaccharide-associated communities we observed initially. However, small-scale changes such as the loss of *Fibrobacter* in MCC when vitamins/cholesterol were removed provide compelling reasons to study the influence of dietary micronutrients on the gut microbiome in future work.

We hypothesize that highly purified synthetic diets enabled microbial ‘specialists’ to bloom beyond their former constraints in the whole-food diets. The high homogeneity of purified fibers may allow these microbes to grow rapidly without needing to wait for the release of pure polysaccharides from cell-matrix degraders, thereby reducing gut microbiome stability. The purified nutrients used in this study differ from “whole foods” in two primary ways: macro/micromolecular composition, and physical accessibility to bacterial degradation. Compositionally, the whole-food diets used in Tinker and Ottesen (2016) were mostly natural foods that, while highly biased in macronutrients, may have had a more diverse nutritional profile according to the “eye” of a bacterium (Hernot et al., 2008; Centanni et al., 2017; Puhlmann and de Vos, 2022). For example, whole and white wheat flour are composed predominantly of endosperm-derived starch but also contain portions of bran and germ, which have structural polysaccharides and bioactive phytochemicals that are targeted by gut bacteria and influence health parameters of the host (Adom et al., 2005; Okarter and Liu, 2010; Cardona et al., 2013; Parkar et al., 2013). Honey contains a complex mixture of sugars (glucose, fructose, disaccharides) and fructooligosaccharides (FOS) in addition to organic acids, nitrogenous compounds, vitamins, and bee-derived enzymes (Sultana et al., 2022). Tuna and butter are similarly high-complexity substrates that offer resident gut microbes diverse metabolizable compounds that are lost in purified dietary components such as those utilized in our study. The purified components used in our synthetic diets are not entirely homogeneous (casein and peptone were used rather than individual amino acids), but as lab-quality reagents, their extraction methods remove the bioactive compounds present basally in the source material, while all whole-food diets retain some of their source material complexity (Puhlmann and de Vos, 2022). Another factor that may contribute to the differences observed between whole and synthetic foods could be the level of processing prior to host feeding. The flours in particular underwent more processing than the other whole foods due to milling, which is known to influence microbial adhesion depending on the resultant particle size (Fernando et al., 2012; Lin et al., 2019). However, even these diets did not shift the gut community composition in the original study (Tinker and Ottesen, 2016), while the starch diet used in this study heavily enriched for an unclassified *Ruminococcaceae* (**Figure 5A**). Both the flours and the starch synthetic diet contained approximately 70% starch by weight and were both finely ground substrates, yet the flours did not contain a single ASV with a relative abundance greater than 3% compared to the starch-associated *Ruminococcaceae* relative abundance of 15%. Although the short 16S rRNA gene region used here only starch-enriched cockroach gut ASVs at the family level, human-associated *Ruminococcus bromii* are established as effective degraders of resistant starch that distribute released glucose rather than utilize it themselves (Abell et al., 2008; Ze et al., 2012; Sun et al., 2016; Rangarajan et al., 2022). The extent to which the starch-associated *Ruminococcaceae* bloomed suggests strong selective enrichment of starch-specialized microbes, although a similar glucose cross-feeding relationship may explain in part why gut community alpha diversity remained high in the starch diet relative to the xylan diet.

Our hypothesis that synthetic diets with purified fibers reduce the need for cooperative metabolism of dietary fiber is supported by our microbial co-occurrence network analysis (**Figure 6**), where the whole-food network was highly interconnected with numerous significant co-occurrence relationships between ASVs, while the synthetic diet network was easily fragmented into modules of microbes that were weakly or negatively associated with the other network members (**Supplementary Figures S13, S14**). Under this hypothesis, when compared to the rich landscape of intrinsic fibers found in whole foods, synthetic diets contain simpler macromolecular structures that may streamline the microbial enzymatic processes of fiber catabolism. This reduction of enzymatic requirements may in turn enable individual fiber specialists, who possess all or most of the necessary machinery, to metabolize large amounts of these purified fibers without aid from other microbes. While some direct cross-feeding relationships are expected to remain, the loss of metabolite diversity may fragment the more nebulous cross-feeding relationships, therefore pruning the number of significant co-occurrence relationships among gut microbes to produce the network presented in this study.

A key limitation of this study is the fact that the comparison group of “whole food”-fed cockroaches was from an earlier experiment and we lacked contemporaneous controls fed whole-food diets. However, an examination of cohort effects suggested that observed responses to synthetic diets were highly conserved across cohorts in experiments conducted 1 year apart (**Figure 3**). Additional caveats to this work regard the purified components used. The original source of a compound can be difficult to identify and may impact the fine structure of the compound despite it appearing comparable to one from a different source. Our source of starch, for example, was derived from potato, which produces higher resistant starch levels than other starches (Patterson et al., 2020). The xylan used in this study is highly soluble and may produce a different gut community than if we had used oat or birch xylan. While these caveats limit some of the conclusions that can be formed, they further highlight the utility of cockroaches in these studies.

Overall, this study showed that synthetic diets that were highly enriched in a single polysaccharide can produce divergent gut microbiome compositions in the American cockroach, which has previously been shown to be highly resistant to diet-induced differences in gut microbiome composition (Tinker and Ottesen, 2016). The individual polysaccharides featured in the different synthetic diets were associated with diet-specific ‘blooms’ of native Firmicutes and Bacteroidota rather than the introduction of new microbial specialists into the community. The enrichment of these ASVs led to fragmented gut microbiota co-occurrence networks with increased inter-individual variability among insects. Together, these results suggest that overconsumption of a single, purified class of polysaccharides can have destabilizing effects on cockroach gut microbiota. This work highlights the use of omnivorous cockroaches and synthetic diets as an *in vivo* enrichment culture system to pinpoint microbial responses to highly processed dietary ingredients while remaining within the context of a host-microbe system, thus facilitating the isolation and improved characterization of novel gut symbionts that are passed over in traditional benchtop microbiology. Future work will examine the functional and metabolic basis of these alternate microbial community compositions and will further explore the ways in which diet complexity and composition impacts gut microbiome homeostasis.

## Data Availability

The datasets presented in this study can be found in online repositories. The names of the repository/repositories and accession number(s) can be found below: https://www.ncbi.nlm.nih.gov/, PRJNA1096047; https://www.ncbi.nlm.nih.gov/, PRJNA1105088; https://www.ncbi.nlm.nih.gov/, PRJNA320546.
