# Supplementary material for: Purified fibers in chemically defined synthetic diets destabilize the gut microbiome of an omnivorous insect model
Source: Front Microbiomes. 2024 Dec 12;3:1477521. doi: 10.3389/frmbi.2024.1477521 (PMC11925550; doi:10.3389/frmbi.2024.1477521)
Supplement: Supplementary file 2 [file Image1.pdf]

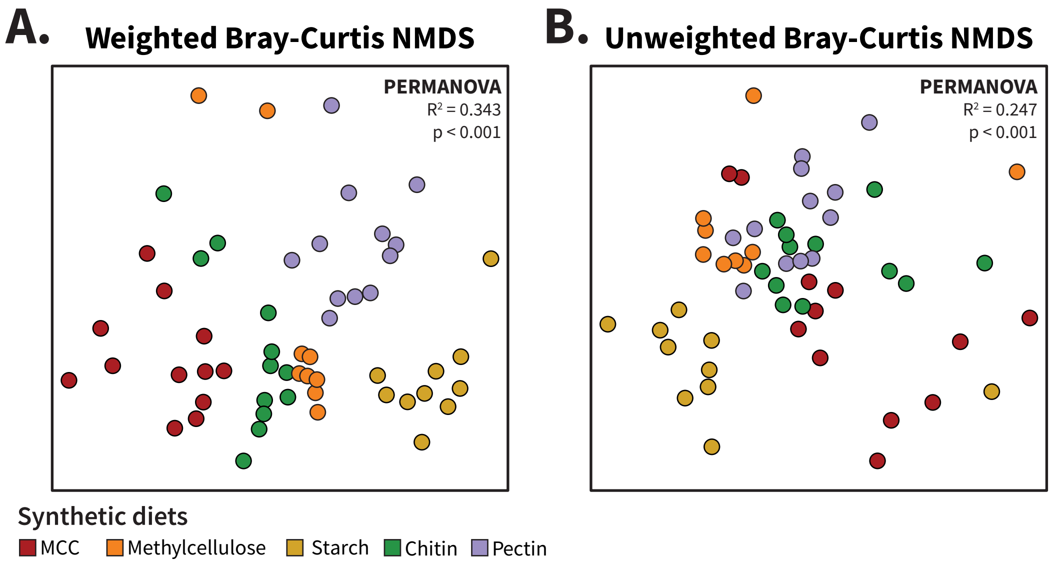

Supplement 1: NMDS ordination analysis of synthetic diet samples excluding xylan-fed cockroaches. As in Figure 1, samples were rarefied a constant depth of 7924 sequences for alpha and beta diversity calculations. Non-metric multidimensional scaling (NMDS) was used to plot **(A)** weighted and **(B)** unweighted Bray-Curtis dissimilarity of gut communities from synthetic diets, excluding those fed the xylan diet. Multivariate analysis was performed using PERMANOVA.
