# Supplementary material for: Purified fibers in chemically defined synthetic diets destabilize the gut microbiome of an omnivorous insect model
Source: Front Microbiomes. 2024 Dec 12;3:1477521. doi: 10.3389/frmbi.2024.1477521 (PMC11925550; doi:10.3389/frmbi.2024.1477521)
Supplement: Supplementary file 3 [file Image2.pdf]

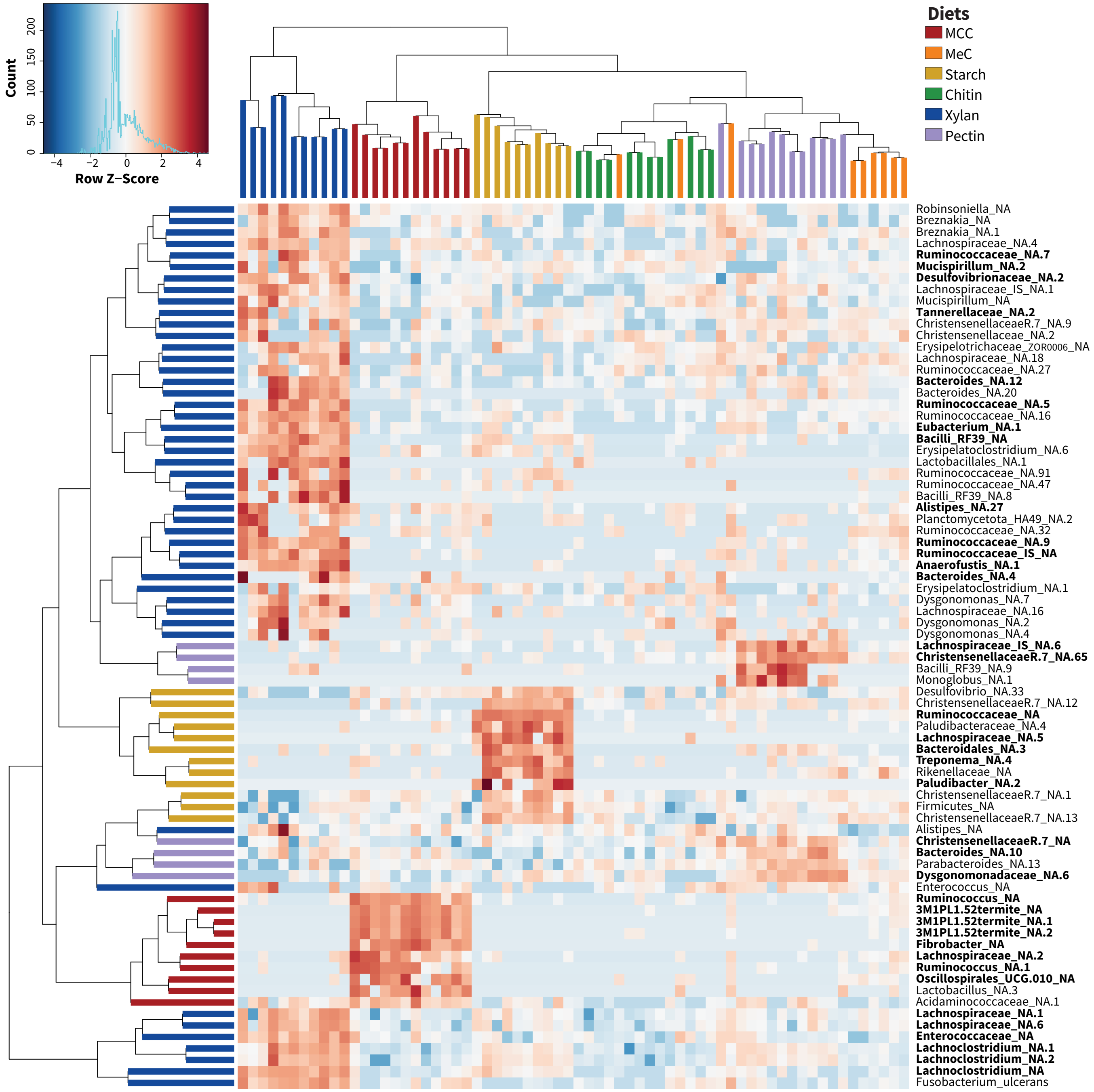

Supplement 2: Diet-characteristic ASVs determined by DESeq2. Pairwise DESeq2 analysis was performed on filtered (present in 5 or more samples) raw counts for all synthetic diets (n=66), and ASVs significant in one diet vs all others at adjusted $p < 0.05$ with a baseMean higher than 10 were selected (76 total). Variance stabilized transformed counts obtained from DESeq2 were scaled by row to generate z-scores for plotting. Dendrograms are colored by diet per sample (column) and diet the ASVs are associated with (row). Bolded names indicate the ASV belongs to “Set 1” as presented in Figure 4.
