# Supplementary material for: Purified fibers in chemically defined synthetic diets destabilize the gut microbiome of an omnivorous insect model
Source: Front Microbiomes. 2024 Dec 12;3:1477521. doi: 10.3389/frmbi.2024.1477521 (PMC11925550; doi:10.3389/frmbi.2024.1477521)
Supplement: Supplementary file 4 [file Image3.pdf]

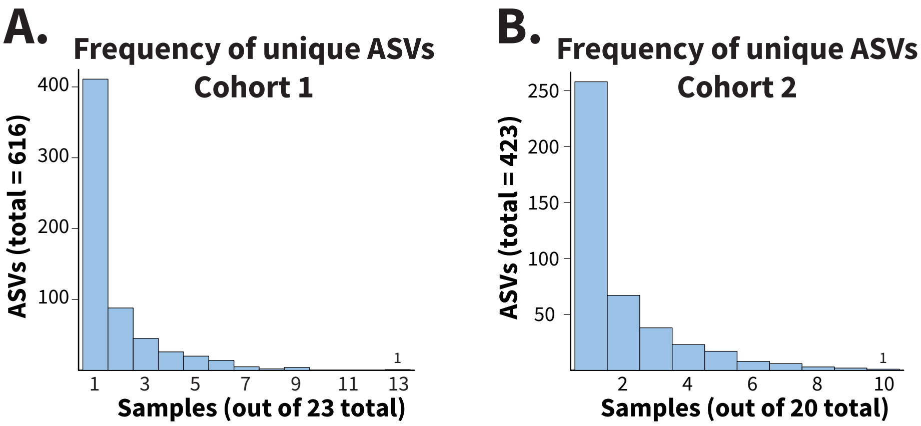

Supplement 3 : Most unique ASVs in the Xylan/MCC replicate experiments are singletons. Unique ASVs from the rarefied **(A)** Cohort 1 and **(B)** Cohort 2 data were assessed for frequency of occurrence using the histogram function in R. Samples were rarefied to 9685 ASVs for comparison.
