# Supplementary material for: Purified fibers in chemically defined synthetic diets destabilize the gut microbiome of an omnivorous insect model
Source: Front Microbiomes. 2024 Dec 12;3:1477521. doi: 10.3389/frmbi.2024.1477521 (PMC11925550; doi:10.3389/frmbi.2024.1477521)
Supplement: Supplementary file 5 [file Image4.pdf]

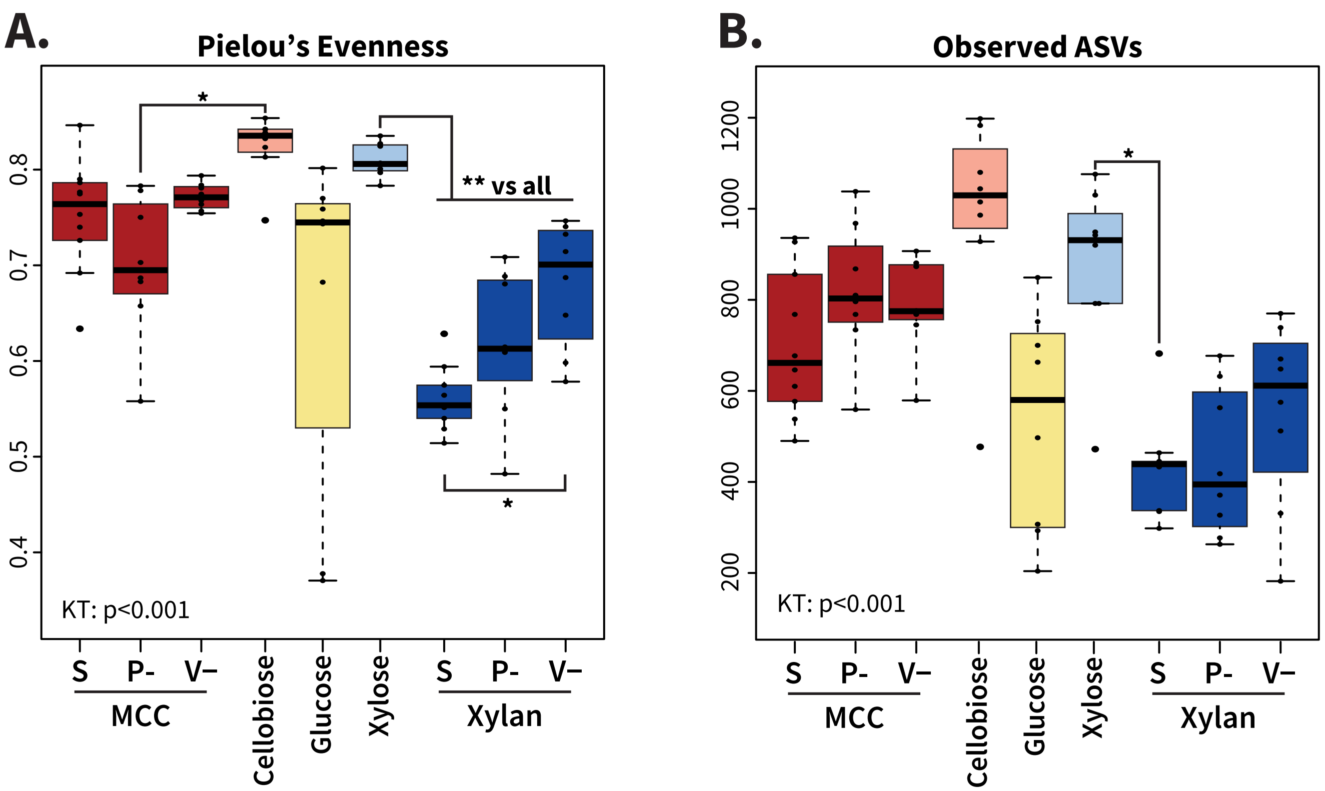

Supplement 4: Additional alpha diversity measures of standard, deficient, and simple-sugar diets. Samples were rarefied a constant depth of 12274 sequences for alpha diversity calculations **(A)** Pielou's evenness and **(B)** number of observed ASVs. MCC: microcrystalline cellulose; S: standard diet; P-: protein-deficient; V-: vitamin-deficient. *p < 0.05; **p<0.01
