# Supplementary material for: Purified fibers in chemically defined synthetic diets destabilize the gut microbiome of an omnivorous insect model
Source: Front Microbiomes. 2024 Dec 12;3:1477521. doi: 10.3389/frmbi.2024.1477521 (PMC11925550; doi:10.3389/frmbi.2024.1477521)
Supplement: Supplementary file 6 [file Image5.pdf]

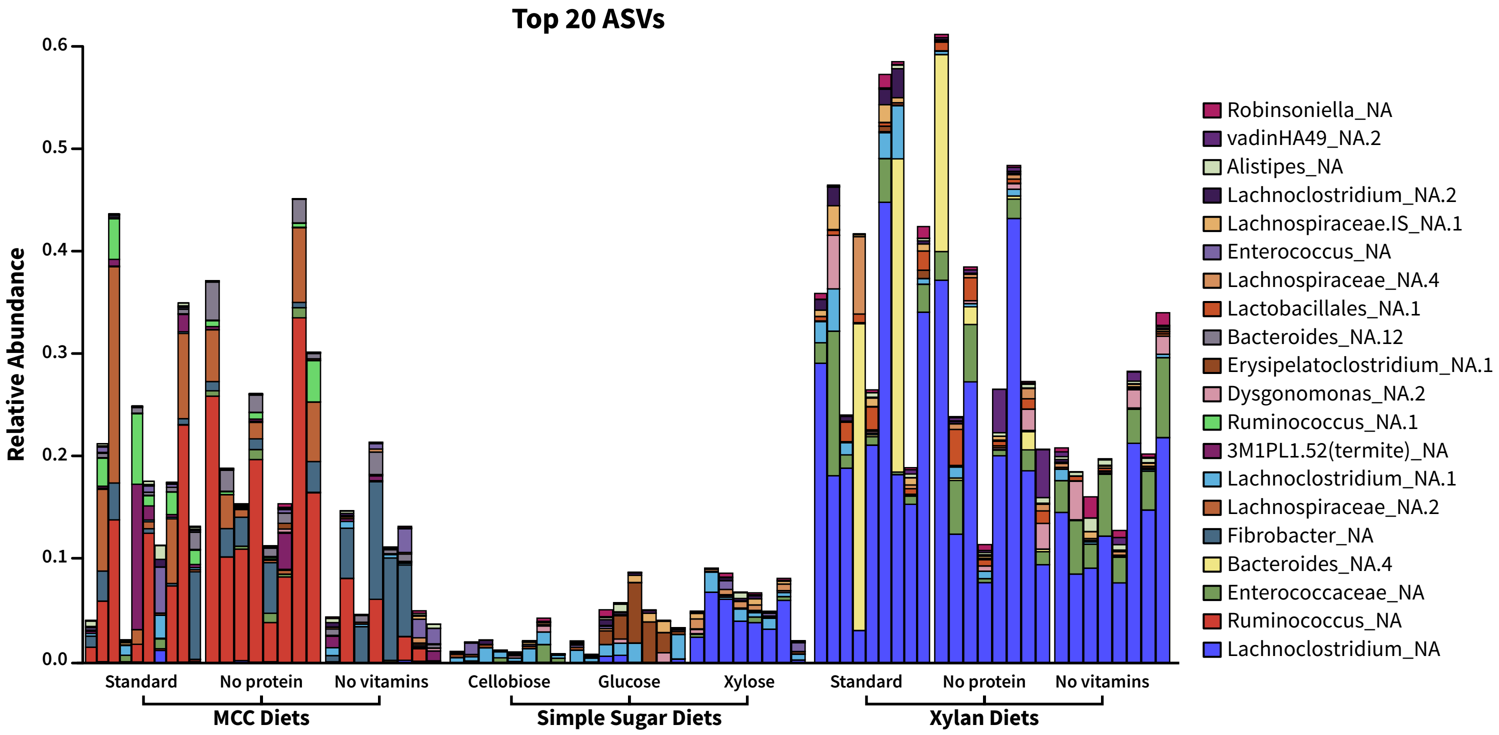

Supplement 5: Relative abundance of top ASVs in xylan/MCC diet variations. ASV tables were converted to proportion tables and sorted from most to least abundant ASV across standard, deficient, and simple-sugar variations of the xylan and MCC diets. The top 20 ASVs were included for visualization.
