# Supplementary material for: Purified fibers in chemically defined synthetic diets destabilize the gut microbiome of an omnivorous insect model
Source: Front Microbiomes. 2024 Dec 12;3:1477521. doi: 10.3389/frmbi.2024.1477521 (PMC11925550; doi:10.3389/frmbi.2024.1477521)
Supplement: Supplementary file 7 [file Image6.pdf]

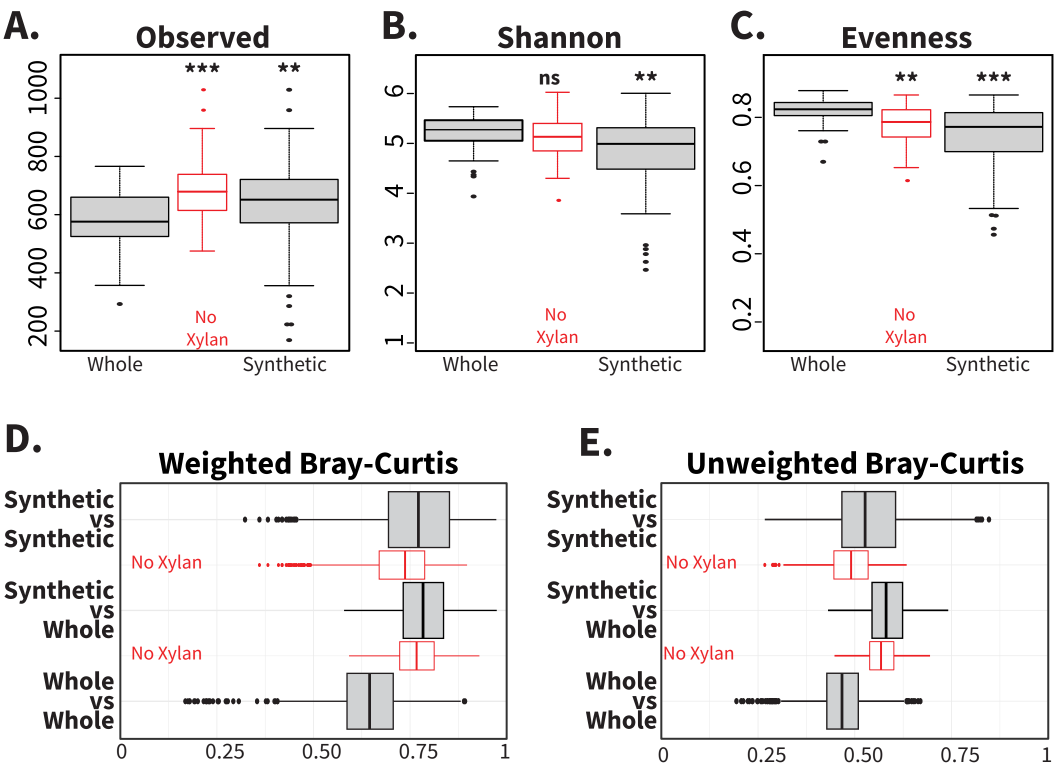

Supplement 6: Overview of alpha and beta diversity differences between whole food and synthetic diets. Raw sequence data from Tinker and Ottesen (2016) were reprocessed using the methods in this experiment to generate comparable ASVs. All samples were rarefied to 7924 ASVs for alpha and beta diversity analysis. Boxplots show **(A)** observed ASVs, **(B)** Shannon index, and **(C)** Pielou's evenness for each diet type, with red boxes representing the synthetic diets minus xylan-fed samples. Wilcoxon rank-sum test was used for pairwise statistical analysis between diet types. Beta diversity boxplots of **(D)** weighted and **(E)** unweighted Bray-Curtis dissimilarity, with red boxes representing synthetic diets excluding xylan. ** =p <0.01; *** =p<0.001, ns =no significance
