# Supplementary material for: Purified fibers in chemically defined synthetic diets destabilize the gut microbiome of an omnivorous insect model
Source: Front Microbiomes. 2024 Dec 12;3:1477521. doi: 10.3389/frmbi.2024.1477521 (PMC11925550; doi:10.3389/frmbi.2024.1477521)
Supplement: Supplementary file 8 [file Image7.pdf]

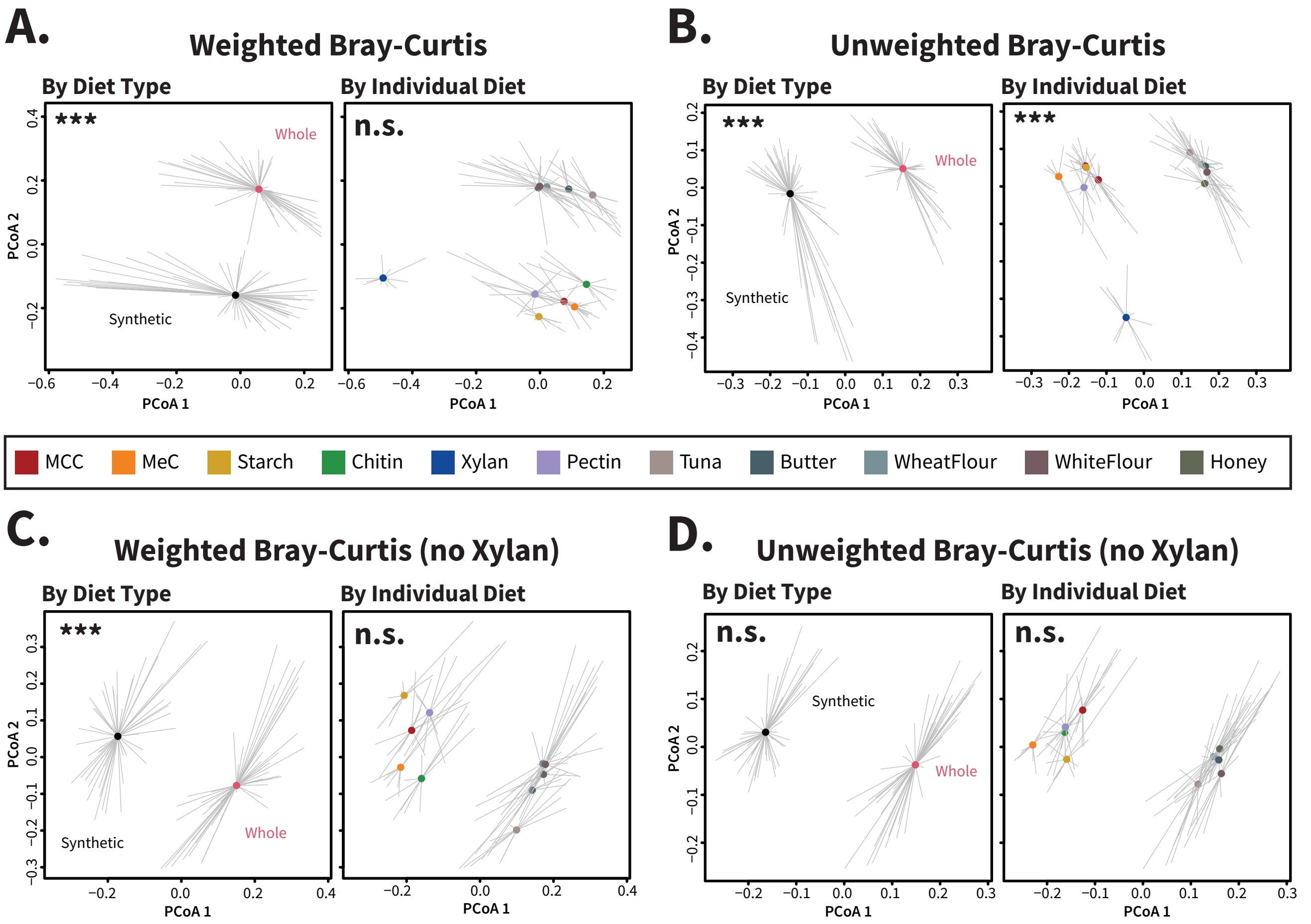

Supplement 7: Beta dispersion analysis of whole food and synthetic diets, with and without xylan-fed samples. The dispersion of **(A,C)** weighted and **(B,D)** unweighted Bray-Curtis dissimilarities was assessed for both diet group and individual diets, both **(A,B)** with and **(C,D)** without xylan-fed samples included in analysis. All samples were rarefied to 7924 ASVs for beta dispersion analysis, and ANOVA statistical testing was used to evaluate if dispersion differed between groups/diets. *** =p<0.001, ns =no significance
