# Supplementary material for: Purified fibers in chemically defined synthetic diets destabilize the gut microbiome of an omnivorous insect model
Source: Front Microbiomes. 2024 Dec 12;3:1477521. doi: 10.3389/frmbi.2024.1477521 (PMC11925550; doi:10.3389/frmbi.2024.1477521)
Supplement: Supplementary file 9 [file Image8.pdf]

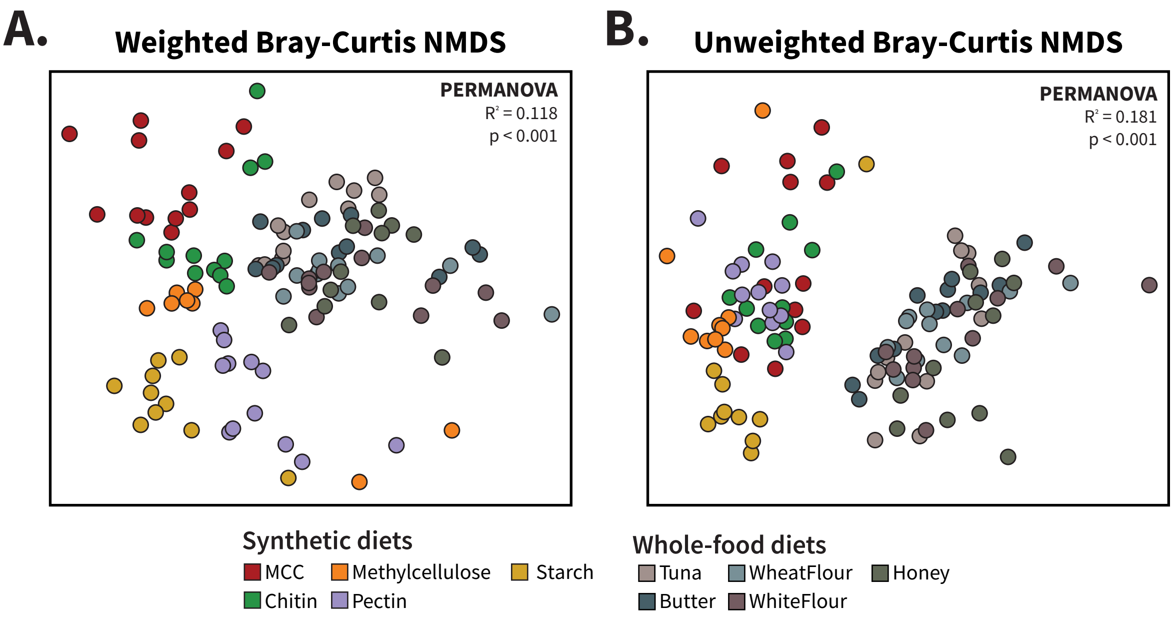

Supplement 8: Differences in beta diversity between whole food and synthetic diets without xylan-fed samples. NMDS ordinations of **(A)** weighted and **(B)** unweighted Bray-Curtis distances between cockroach hindgut samples from this study ("Synthetic") and data from Tinker and Ottesen (2016) for cockroaches fed tuna, butter, honey, wheat flour, and white flour ("Whole Food Diets"), excluding xylan-fed samples. Samples were rarefied to 7924 ASVs for beta diversity analysis. R^2 and p-values for diet type comparisons were calculated using PERMANOVA.
