# Supplementary material for: Purified fibers in chemically defined synthetic diets destabilize the gut microbiome of an omnivorous insect model
Source: Front Microbiomes. 2024 Dec 12;3:1477521. doi: 10.3389/frmbi.2024.1477521 (PMC11925550; doi:10.3389/frmbi.2024.1477521)
Supplement: Supplementary file 10 [file Image9.pdf]

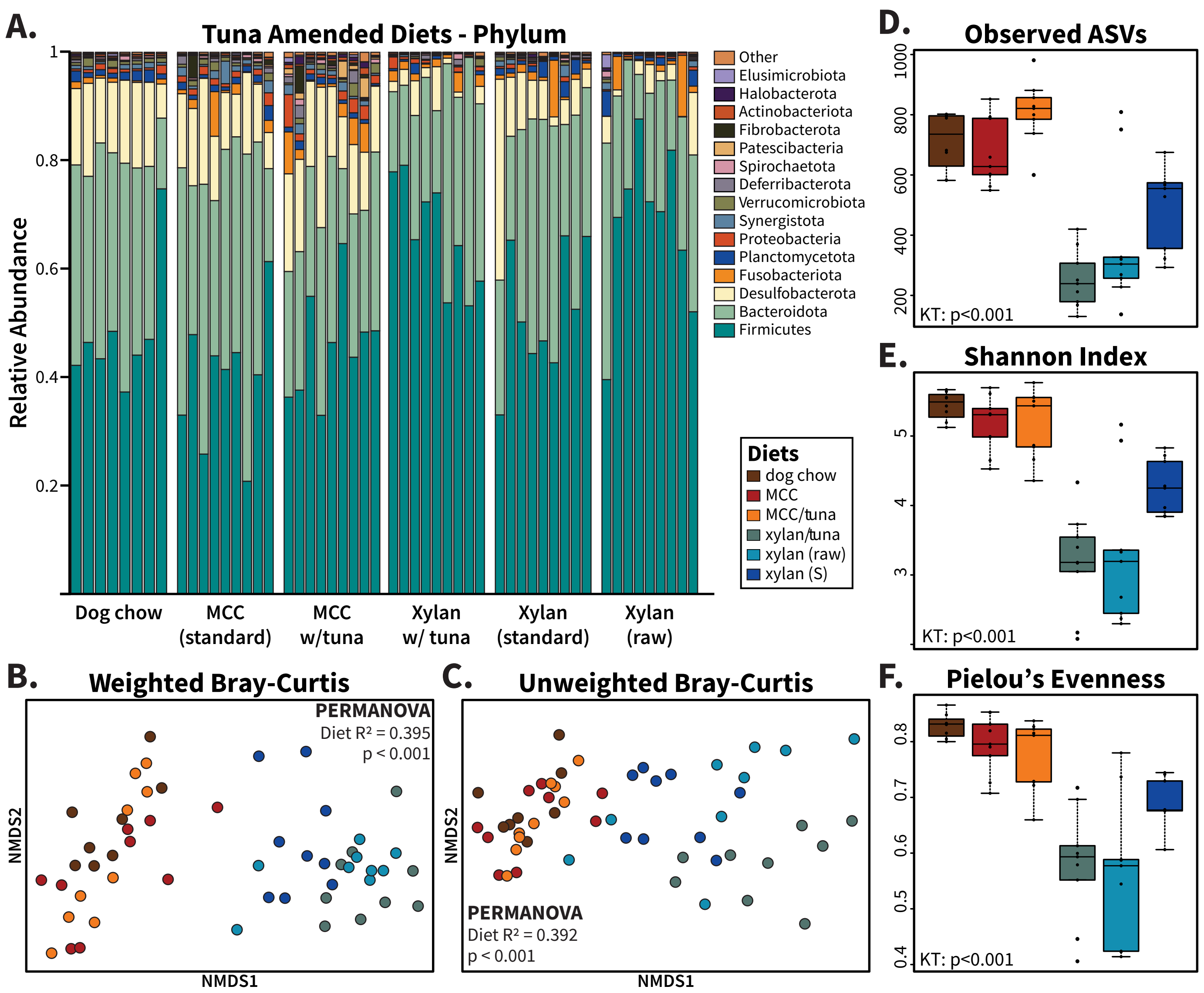

Supplement 9: Complex proteins did not reduce the influence of fiber source on gut microbiome composition. Samples were rarefied a constant depth of 12274 sequences for alpha and beta diversity calculations, while relative abundance was used to visualize phylum-level composition. **(A)** depicts phyla relative abundance. NMDS ordinations were generated for **(B)** weighted and **(C)** unweighted Bray-Curtis dissimilarity and assessed for significance with PERMANOVA. Alpha diversity measures **(D)** observed ASVs, **(E)** Shannon index, and **(F)** Pielou's evenness are plotted with boxplots and statistics were calculated using Kruskal test. MCC: microcrystalline cellulose; S: standard diet; KT: Kruskal test
