# Supplementary material for: Purified fibers in chemically defined synthetic diets destabilize the gut microbiome of an omnivorous insect model
Source: Front Microbiomes. 2024 Dec 12;3:1477521. doi: 10.3389/frmbi.2024.1477521 (PMC11925550; doi:10.3389/frmbi.2024.1477521)
Supplement: Supplementary file 12 [file Image11.pdf]

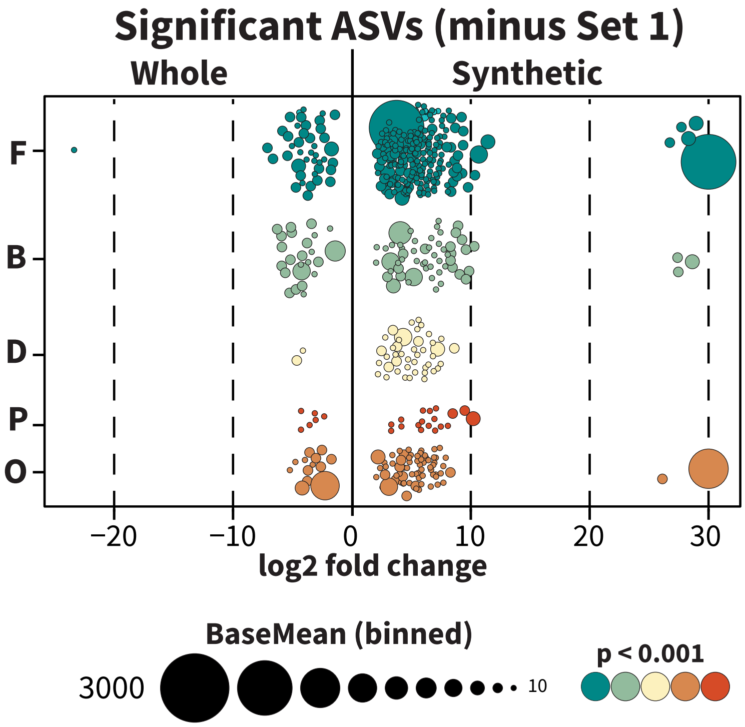

Supplement 11: Significantly enriched ASVs between whole food and synthetic diets, excluding Set 1 ASVs. Raw sequence count tables of whole food and synthetic diets were filtered to include ASVs present in at least 5 samples (out of 125 total) and analyzed using DESeq2 with diet type as the design factor. ASVs identified as significant ($p < 0.001$), excluding Set 1, are visualized.
