# Supplementary material for: Purified fibers in chemically defined synthetic diets destabilize the gut microbiome of an omnivorous insect model
Source: Front Microbiomes. 2024 Dec 12;3:1477521. doi: 10.3389/frmbi.2024.1477521 (PMC11925550; doi:10.3389/frmbi.2024.1477521)
Supplement: Supplementary file 13 [file Image12.pdf]

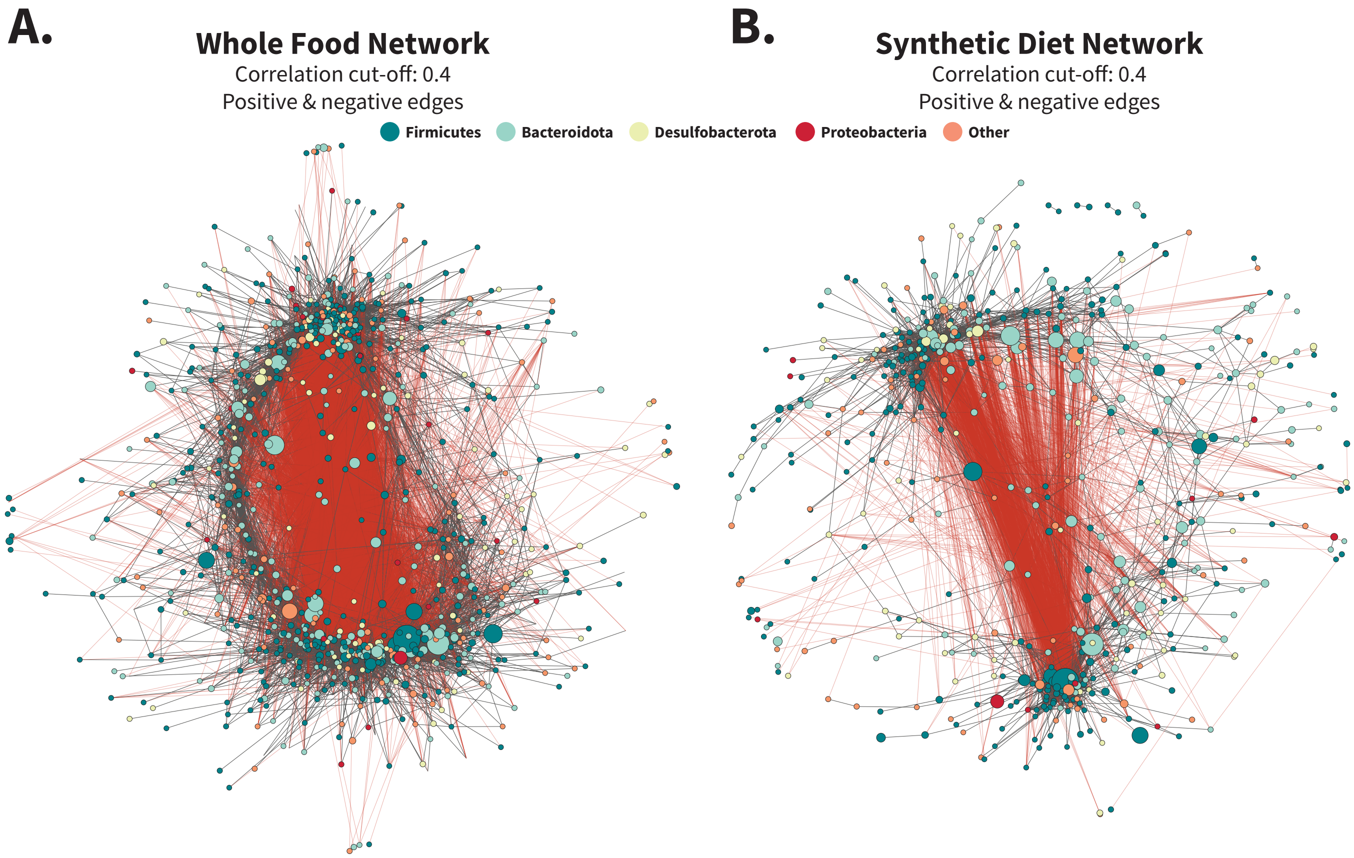

Supplement 12: Whole food and synthetic networks including negative edges. Networks were calculated by SparCC from filtered count tables for **(A)** whole food and **(B)** synthetic diets separately to create two distinct networks. Count tables were filtered to include only ASVs present in at least 25% of the samples per diet set, resulting in 976 ASVs for whole food diets and 700 for synthetic. Networks were further pruned to remove edges with absolute values smaller than 0.4 before exporting to Cytoscape. Negative edges were included during initial layout generation with the edge-weighted spring embedded layout method.
