# Supplementary material for: Purified fibers in chemically defined synthetic diets destabilize the gut microbiome of an omnivorous insect model
Source: Front Microbiomes. 2024 Dec 12;3:1477521. doi: 10.3389/frmbi.2024.1477521 (PMC11925550; doi:10.3389/frmbi.2024.1477521)
Supplement: Supplementary file 14 [file Image13.pdf]

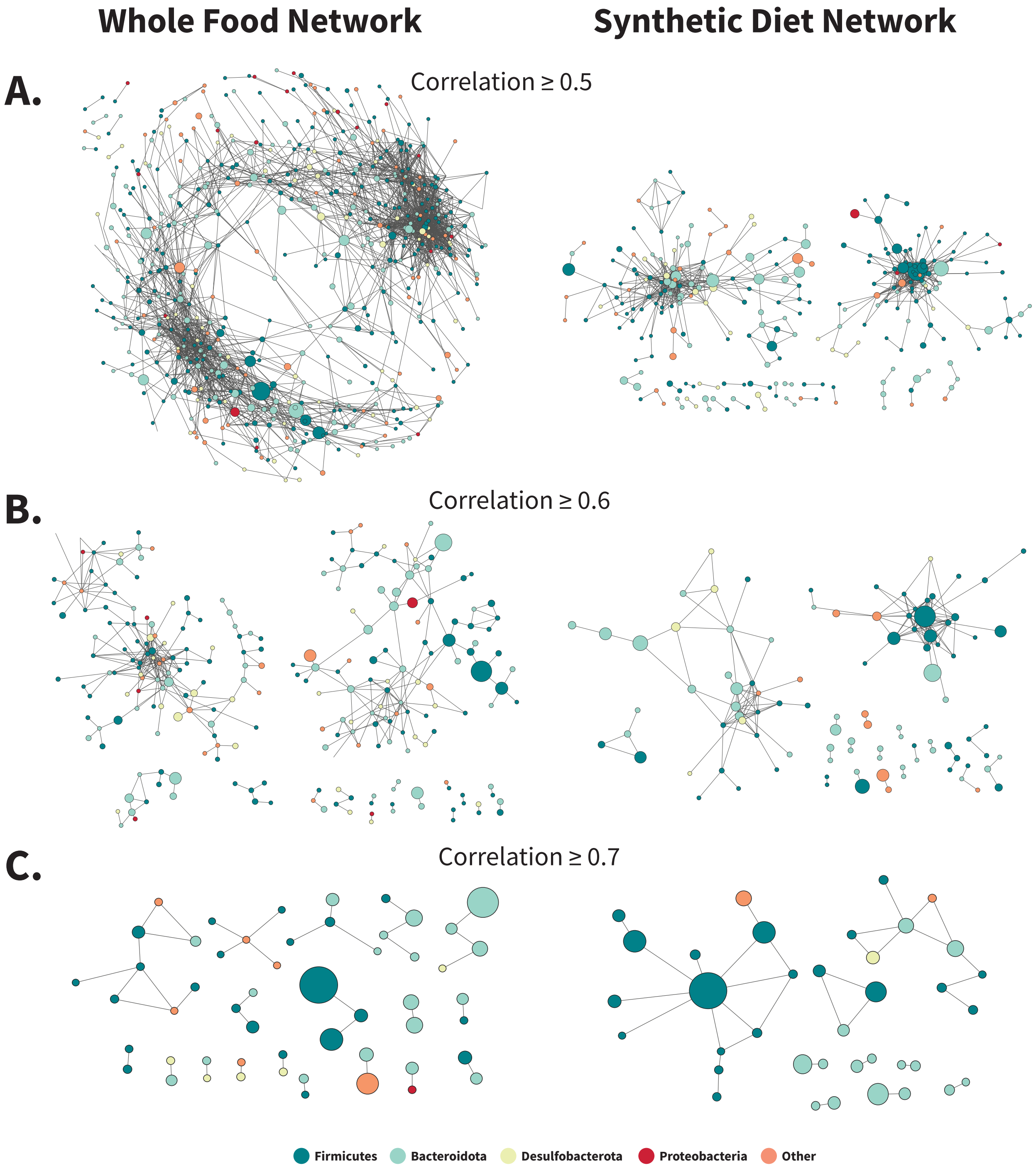

Supplement 13: Whole food and synthetic networks at higher correlation cut-off levels. Networks generated for Figures 6 and S11 were filtered in Cytoscape by edge weight to visualize networks at **(A)** 0.5, **(B)** 0.6, and **(C)** 0.7 SparCC correlation values, with isolated nodes removed if they lost all adjacent edges. Data on edges, nodes, and connected components at these cut-off levels are presented in Figure 6C-E.
