# Supplementary material for: Purified fibers in chemically defined synthetic diets destabilize the gut microbiome of an omnivorous insect model
Source: Front Microbiomes. 2024 Dec 12;3:1477521. doi: 10.3389/frmbi.2024.1477521 (PMC11925550; doi:10.3389/frmbi.2024.1477521)
Supplement: Supplementary file 15 [file Image14.pdf]

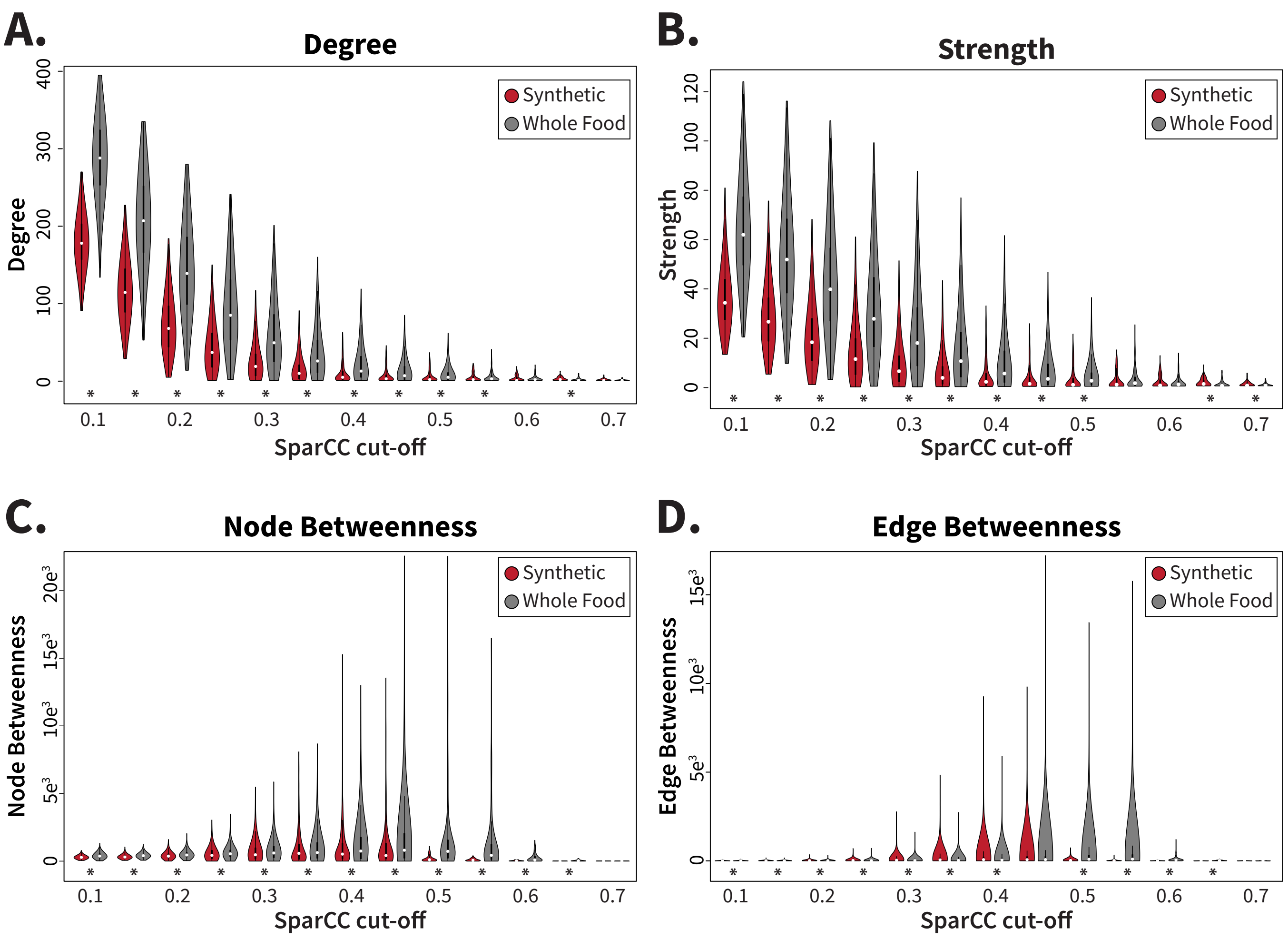

Supplement 14: Whole food diets encourage more interconnected network formation than synthetic diets. SparCC networks were generated for whole food and synthetic diet types using raw ASV counts excluding uncommon taxa (< 25% of samples in diet type). The networks were analyzed at increasing levels of positive associations to compare **(A)** degrees per node, **(B)** strength of co-correlations per node, **(C)** node betweenness, and **(D)** edge betweenness. Statistics were calculated using the Wilcoxon rank-sum test. * = $p<0.05$ Red: Synthetic; Grey: Whole Food.
