# Supplementary material for: Purified fibers in chemically defined synthetic diets destabilize the gut microbiome of an omnivorous insect model
Source: Front Microbiomes. 2024 Dec 12;3:1477521. doi: 10.3389/frmbi.2024.1477521 (PMC11925550; doi:10.3389/frmbi.2024.1477521)
Supplement: Supplementary file 16 [file Table1.docx]

| **Cohort** | **Diet** | **Individuals dissected** | **Sequenced**  total (male/female) | **Raw reads**  **(total / average)** | **Quality reads**  **(total / average)** |
| --- | --- | --- | --- | --- | --- |
| **1**  ***S****: 2/2020*  ***E:*** *3/2020* | MCC-1 | 12 | 12 ( 6 / 6 ) | 1,029,110 / 85,759 | 823,707 / 68,642 |
|  | Chitin | 12 | 12 ( 9 / 3 ) | 310,657 / 25,888 | 2726,57 / 22,721 |
|  | Pectin | 12 | 12 ( 9 / 3 ) | 231,967 / 19,331 | 203,208 / 16,934 |
|  | Xylan-1 | 12 | 12 ( 5 / 7 ) | 568,859 / 47,405 | 417,805 / 34,817 |
|  | Methylcellulose | 12 | 12 ( 8 / 4 ) | 587,125 / 48,927 | 462,819 / 38,568 |
| **2**  ***S:*** *11/2020*  ***E:*** *11/2020* | Starch* | 12 | 10 ( 9 / 1 ) | 488,500 / 48,850 | 296,205 / 29,621 |
|  | MCC-2 | 12 | 10 ( 8 / 2 ) | 613,777 / 61,378 | 340,395 / 34,040 |
|  | Xylan-2 | 12 | 10 ( 9 / 1 ) | 1,078,499 / 107,850 | 736,808 / 73,681 |
| **3**  ***S:*** *1/2022*  ***E:*** *2/2022* | Xylan P- | 12 | 8 ( 4 / 4 ) | 556,764 / 69,596 | 444,612 / 55,577 |
|  | Xylan V- | 12 | 8 ( 4 / 4 ) | 692,806 / 86,601 | 556,169 / 69,521 |
|  | MCC P- | 12 | 8 ( 4 / 4 ) | 680,675 / 85,084 | 582,030 / 72,754 |
|  | MCC V- | 12 | 8 ( 4 / 4 ) | 575,169 / 71,896 | 494,232 / 61,779 |
|  | Xylose | 12 | 8 ( 4 / 4 ) | 655,253 / 81,907 | 556,133 / 69,517 |
|  | Cellobiose | 12 | 8 ( 4 / 4 ) | 527,794 / 65,974 | 409,609 / 51,201 |
|  | Glucose | 12 | 8 ( 4 / 4 ) | 450,316 / 56,290 | 374,747 / 46,843 |
| **4^#^**  ***S:*** *8/2023*  ***E:*** *9/2023* | MCC-3 | 12 | 10 | 370,518 / 37,052 | 278,299 / 27,830 |
|  | Xylan-3 | 12 | 10 | 1,277,470 / 127,747 | 940,769 / 94,077 |
|  | Xylan-3-raw | 12 | 10 | 1,306,817 / 130,682 | 914,751 / 91,475 |
|  | MCC-Tuna | 12 | 10 | 1,753,300 / 175,330 | 1,386,390 / 138,639 |
|  | Xylan-Tuna | 12 | 10 | 652,675 / 65,268 | 436,507 / 43,651 |
|  | Dog chow | 12 | 10 | 257,678 / 25,768 | 190,726 / 19,073 |
| ***Totals*** | *21 diets* | *252* | *206* | 14,665,729 / 72,599 | 10,845,921 */* 55,284 |

* The starch diet was analyzed with cohort 1

# sex was not recorded for cohort 4
