# Supplementary material for: Purified fibers in chemically defined synthetic diets destabilize the gut microbiome of an omnivorous insect model
Source: Front Microbiomes. 2024 Dec 12;3:1477521. doi: 10.3389/frmbi.2024.1477521 (PMC11925550; doi:10.3389/frmbi.2024.1477521)
Supplement: Supplementary file 11 [file Image10.pdf]

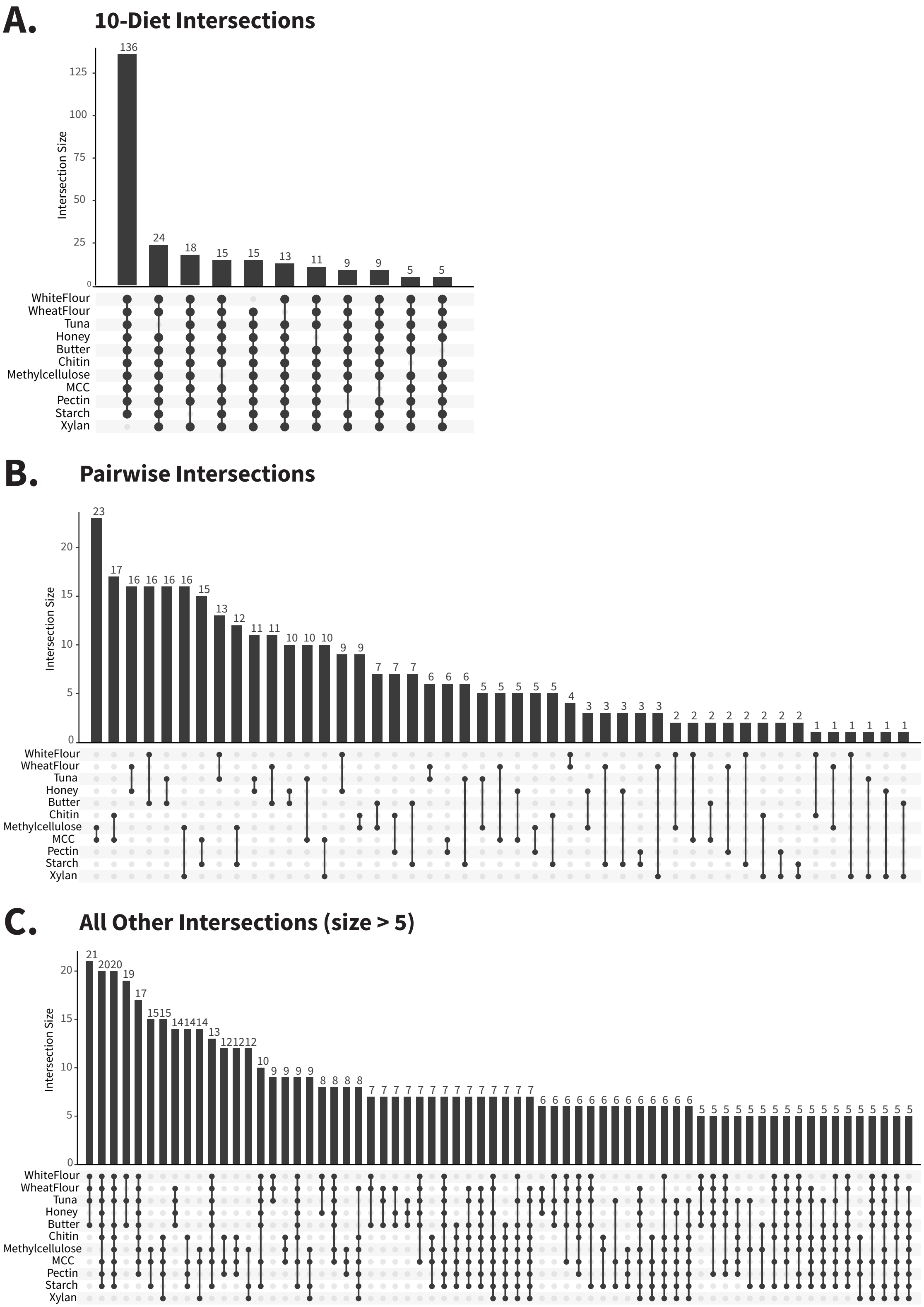

Supplement 10: Additional UpSet plots. The rarefied samples (7924 depth) from whole food and synthetic diets were used to generate UpSet plots. **(A)** depicts sets where ASVs are found in all but one of the diets. **(B)** depicts sets found in only two diets. **(C)** contains remaining sets not yet displayed with at least 5 ASVs. Additional sets were not included.
